# The construction and psychometric evaluation of the self-structure scale: a bidimensional model of self-structure

**DOI:** 10.3389/fpsyt.2026.1942305

**Published:** 2026-09-10

**Authors:** Xinyi Li, Jie Zhong, Sheng-Sheng Fan, Jia-Yi Yin, Xing Gao, Wei Li

**Affiliations:** 1 Beijing Key Laboratory of Brain-Computer Interface and Mental Health Modulation, Key Laboratory of Machine Perception (Ministry of Education), Clinical and Health Psychology Department, School of Psychological and Cognitive Sciences, Peking University, Beijing, China; 2 Department of Psychiatry, Shanxi Bethune Hospital, Shanxi Academy of Medical Sciences, Third Hospital of Shanxi Medical University, Tongji Shanxi Hospital, Taiyuan, China

**Keywords:** narcissism, personality assessment, personality functioning, psychodynamic theory, psychometric validation, scale development, self-psychology, self-structure

## Abstract

**Background:**

Although psychoanalytic and personality theories have consistently highlighted the central role of self-structure, existing psychometric tools predominantly measure isolated personality traits or narcissistic subtypes, with scarce instruments dedicated to evaluating the organizational mechanisms governing self-related functioning. This study puts forward a bidimensional theoretical framework of self-structure and constructs the Scale of Self-Structure (SOSS), aiming to empirically verify the measurable manifestation of the proposed theoretical dimensions.

**Methods:**

Two sequential empirical studies were performed to construct and psychometrically validate the SOSS. Study 1 covered item pool development, exploratory factor analysis (EFA), internal consistency testing, and test–retest reliability verification. A total of 1,954 participants were recruited for initial scale refinement, and an independent subsample of 128 respondents completed retest assessments to evaluate temporal stability. Study 2 recruited 3,805 participants split across two separate cohorts: Sample 3 (n = 2,570) and Sample 4 (n = 1,235). This sample was used for cross-sample structural validation and comprehensive psychometric examinations, including confirmatory factor analysis (CFA), exploratory structural equation modeling (ESEM), internal reliability evaluation, multi-group measurement invariance tests, discriminant validity estimation, and person-centered latent profile clustering. Supplementary narcissism measurement data collected from Sample 4 further supported analyses of convergent and nomological validity.

**Results:**

EFA yielded a 28-item SOSS encompassing four correlated first-order factors: Positive Self, Self-Center, Idealized Identification, and Vulnerable Self. In Study 2, competing measurement models were compared. The four-factor exploratory structural equation modeling (ESEM) solution exhibited the best fit to the data, implying that self-structural processes consist of interrelated yet partially overlapping components, rather than following a strictly hierarchical structure. The SOSS showed acceptable internal consistency and temporal stability, supported measurement invariance across gender, and displayed adequate discriminant validity together with theoretically consistent correlations with grandiose and vulnerable narcissism. Subsequent person-centered analyses tentatively revealed four distinct profile configurations within the two-dimensional structural space defined by the SOSS.

**Conclusions:**

This study provides preliminary empirical evidence supporting the SOSS as a multidimensional instrument for assessing individual differences in self-structural organization. Established based on psychodynamic self-organization theories and validated through systematic psychometric examinations, the SOSS may serve as a tentative framework for understanding self-investment, relational functioning, and self-cohesion beyond traditional trait-based indicators. Given the nonclinical and culturally homogeneous sample of the present study, future investigations incorporating longitudinal designs, demographically diverse cohorts, clinical populations, and cross-cultural validation are required to further elucidate the developmental implications, generalizability, and clinical applicability of the self-structural dimensions captured by the SOSS.

## 1 Introduction

### 1.1 Conceptualizing the self and self-structure

Psychoanalytic theory has long regarded the organization of the self as a central construct in understanding human subjectivity, personality maturation, and psychopathological formation (1, 2). While Freud’s topographical and structural models offered foundational interpretations of psychic systems, mental agencies, and intrapsychic conflicts, they did not fully conceptualize the self as an experiential and hierarchically organizing subjective entity (3). Subsequent theoretical advancements in ego psychology, object relations theory, and self-psychology further distinguished the self from basic ego functions and discrete self-representations. These frameworks conceptualize the self as a higher-order organizational core that synthesizes personal experiences, affective states, core values, and self and object representations to form a relatively unified and coherent subjective worldview (4–8).

Within this theoretical tradition, the self is conceived not merely as an aggregate of self-referential beliefs, but as a dynamic organizational system that encodes personal experiences, sustains emotional investment, and maintains temporal continuity of individual identity. Autobiographical memories, affective connotations, and multifaceted self-representations collectively shape a stable sense of selfhood and preserve psychological coherence across changing situations and environments (8–11). This integrative theoretical perspective is particularly pivotal to psychodynamic and personality research, as disruptions in self-coherence, self-stability, and self-regulatory capacity have been well-documented to correlate closely with personality pathology, particularly narcissistic and borderline personality configurations (12–14).

Grounded in these theoretical foundations, the present study defines self-structure as the relatively enduring yet modifiable organizational pattern that governs the linkage and regulation of self-relevant experiences, affective states, self-representations, and personal values (15, 16). Serving as the core psychological scaffold of individual subjectivity, self-structure shapes individuals’ interpretation of life events, meaning attribution, maintenance of self-continuity, and interpersonal relational patterns (2, 13). Its development reflects both innate constitutional dispositions and interpersonal relational experiences, with early caregiving environments and internalized object relations exerting a particularly important role on the organization, stabilization, and defensive regulation of the self (17, 18). In this regard, self-structure is conceptually broader than self-concept or personal identity. It encompasses not only explicit self-beliefs, but also the dynamic organizational principles that structure, emotionally invest, and regulate self-relevant meanings across temporal and interpersonal contexts (8, 16, 19). Importantly, theoretical models of self-structure do not necessarily imply a simple psychometric structure in which all observable manifestations of the self are organized into strictly independent hierarchical components. Instead, empirical operationalization requires examination of alternative measurement representations that may capture the complexity and interrelatedness of self-organizational processes.

### 1.2 Narcissism as a phenotypic expression of self-structure

Within the psychoanalytic tradition, narcissism has long served as a central theoretical lens for investigating self-organization and its inherent vulnerabilities. Freud’s seminal formulation originally conceptualized narcissism as libidinal investment in the ego, while Hartmann subsequently clarified the distinction between the ego as a set of psychological functions and the self as the subjective person toward whom such investment is directed (3, 4). This formulation established a foundational connection between narcissistic phenomena and the distribution of psychological investment across the self and external objects. Subsequent psychoanalytic elaborations further delineated both the defensive adaptiveness and developmental functions of narcissism. Kernberg focused on pathological narcissistic organization marked by grandiosity, interpersonal aggressiveness, and impaired object relations, while Kohut reconceptualized narcissism as a fundamental developmental line of the self shaped by mirroring and idealizing selfobject experiences (6, 7, 12, 20). Despite their theoretical differences, these theoretical perspectives converge on the notion that narcissism cannot be reduced to a mere set of symptoms or superficial personality traits; instead, it represents a phenotypic manifestation of the self’s characteristic patterns of emotional investment, structural stabilization, and affective regulation.

Consistent with psychodynamic conceptualizations, contemporary personality research has increasingly conceptualized the multidimensional nature of narcissism, rather than viewing it as a unitary construct. A substantial body of empirical literature supports the distinction between grandiose and vulnerable narcissism (21–24). Grandiose narcissism is predominantly characterized by self-importance, interpersonal entitlement, exhibitionism, dominance striving, and relatively high explicit self-esteem. In contrast, vulnerable narcissism manifests as interpersonal hypersensitivity, pervasive shame, chronic insecurity, contingent self-worth, and prominent internalizing distress (25–29). Importantly, grandiose and vulnerable narcissistic phenotypes are not mutually exclusive categories. They may coexist within the same individual, share common regulatory functions, and fluctuate dynamically across interpersonal contexts and developmental periods (30–33).

These cumulative empirical and theoretical findings suggest that diverse narcissistic presentations can be meaningfully interpreted as phenotypic expressions of underlying self-structural properties. Heuristically analogous to the distinction between genotype and phenotype, observable grandiose, vulnerable, and fluctuating narcissistic patterns may be understood as different expressions of more fundamental organizational characteristics of the self as they interact with developmental and interpersonal conditions (26, 34). This perspective positions narcissism as an empirically tractable window into core self-structural features, including theoretically proposed dimensions related to psychological investment in the self and relational space required for self-maintenance. Accordingly, the present study does not treat narcissism as a standalone research target; instead, it examines theoretically expected associations with narcissistic traits to provide convergent and nomological evidence for the construct validity of the Scale of Self Structure (SOSS).

### 1.3 Existing models and remaining questions

Classical psychoanalytic theories provide foundational frameworks for understanding the relationship between self-organization and narcissistic functioning. Freud’s linear model located self-directed and object-directed love along a single continuum of libidinal continuum, whereas Kohut’s bipolar model proposed that self-development proceeds along grandiose–exhibitionistic and idealizing developmental lines shaped by responsive selfobject experiences (3, 6, 7). These seminal theoretical perspectives have fostered abundant clinical insights and empirical investigations, encompassing inquiries into adaptive and pathological narcissism, the formation of internal selfobjects, and the role of early caregiving environments in shaping internalized object relations that underpin self-organization.

Despite these theoretical advances, several critical issues remain insufficiently resolved. Although the grandiose–vulnerable dichotomy has become a dominant paradigm in modern narcissism research, converging empirical evidence indicates that these phenotypic expressions cannot be fully understood as discrete, stable traits. Individuals frequently display mixed narcissistic features, with their narcissistic presentations dynamically fluctuating across developmental stages, interpersonal contexts, and life events (32, 33, 35). Widely adopted self-report instruments, including the Narcissistic Personality Inventory (NPI) and the Pathological Narcissism Inventory (PNI), have significantly advanced the quantitative assessment of narcissistic traits (23, 36, 37). However, these conventional scales predominantly capture narcissistic characteristics and associated regulatory tendencies, including interpersonal superiority, entitlement, contingent self-worth, shame proneness, and social hypersensitivity. As such, they provide limited direct assessment of the underlying self-structural organizational processes through which the self sustains psychological investment, uses relational experiences for development and affect regulation, and preserves coherence under social frustration, interpersonal threat, and external evaluation.

Contemporary dimensional and spectrum models have further demonstrated the heterogeneous and context sensitivity of narcissistic functioning and its multifaceted developmental outcomes (22, 26, 38–40). In parallel, research on adverse childhood experiences and pathological narcissism has highlighted the importance of early relational environments in shaping later narcissistic vulnerability (41). Collectively, these studies have enriched the understanding of narcissistic phenotypic manifestations, developmental pathways, and individual heterogeneity. Nevertheless, extant research has largely overlooked the underlying self-structural architecture that generates and sustains diverse narcissistic presentations.

This theoretical gap is particularly prominent from a psychodynamic standpoint, which conceptualizes narcissistic phenomena as emergent outcomes of broader self-organizational processes rather than isolated trait symptoms. Recent theoretical work has proposed that this underlying organization can be described along two conceptually distinct yet dynamically interrelated dimensions: the magnitude of psychological investment directed toward the self and the relational and developmental space required for the maintenance and expansion of self-structure (15, 16). These dimensions provide a potential structural account of why similar narcissistic phenotypes may arise from different forms of self-organization, and why grandiose and vulnerable features may coexist or alternate within the same individual. The central methodological challenge is therefore to translate these theoretically derived dimensions into measurable constructs that can be examined within contemporary psychometric and personality research.

In summary, Freud’s theoretical framework primarily emphasized the investment of libidinal energy in the ego (or the self, according to Hartmann’s later conceptualization), whereas Kohut’s theory of narcissistic development conceptualized mirroring and idealizing selfobject experiences as psychological processes through which the self acquires developmental space from the interpersonal environment. Within phenomenological classifications of narcissistic personality disorder (NPD), vulnerable narcissism has been suggested to fundamentally reflect a sense of self-related emptiness, which, from a theoretical perspective, may be associated with the density of libidinal investment directed toward the self (22, 24). In contrast, grandiose narcissism may be conceptualized as reflecting an expansion of psychological self-space. Therefore, the underlying distinctions between vulnerable and grandiose narcissism may represent differences occurring at different levels of self-organization. This perspective provides a potential framework for explaining why these two narcissistic manifestations exhibit both theoretical distinctions and phenomenological overlap. This conceptual framework constitutes the theoretical rationale for introducing the concept of self-density in the present study. Self-density is defined as the density of libidinal energy investment within the psychological developmental space required for the maintenance and expansion of self-structure, and is proposed as a novel perspective for understanding the underlying structure of narcissistic functioning (15, 16). Importantly, self-density is conceptualized as a heuristic theoretical descriptor rather than an independently measurable psychometric dimension. It is used to describe relative patterns of self-organization emerging from the interaction between Self-Cathexis and Self-Space Need, rather than as a directly calculated quantitative index. The present study addresses this challenge by developing and validating a new instrument grounded in a bidimensional model of self-structure.

### 1.4 A bidimensional model of self-structure

Freud’s theoretical framework provides an important starting point for the structural conceptualization of narcissism by locating narcissistic phenomena within the differential distribution of libidinal investment between the self and external objects (3, 4). From this perspective, narcissism reflects, at least in part, the degree of psychological investment directed toward the self. Kohut’s self-psychology extended this formulation by emphasizing that self-development also depends on responsive selfobject experiences, particularly mirroring and idealizing relationships that facilitate the transformation of archaic grandiosity and idealization into stable self-esteem, self-soothing capacities, and adaptive narcissistic functioning (6, 7, 16, 42). Taken together, Freud’s emphasis on self-directed psychological investment and Kohut’s emphasis on relationally mediated self-development suggest that self-organization cannot be adequately described along a single continuum of self-love and object-love. Instead, it requires consideration of both the psychological weight allocated to the self and the relational conditions through which the self is developed, stabilized, and regulated. Although Kohut’s bipolar model substantially broadened the understanding of narcissistic development, it did not explicitly specify the structural relationship between self-investment and selfobject-dependent developmental space, nor did it fully explain how grandiose and vulnerable states can coexist or fluctuate within the same person (7, 43, 44).

Building on these theoretical foundations, the present study proposes a bidimensional theoretical framework of self-structure consisting of two conceptually distinct but dynamically interrelated dimensions: Self-Cathexis and Self-Space need (15, 16). Self-Cathexis refers to the degree to which the self is subjectively experienced as psychologically valuable and worthy of sustained internal investment. Rooted in Freud’s distinction between ego-cathexis and object-cathexis and Hartmann’s differentiation between ego functions and the self, this dimension concerns psychological investment directed toward the integrated subjective self rather than toward ego functioning alone (3, 4). It encompasses enduring self-directed attention, affective care, intrinsic self-regard, and the subjective legitimacy individuals grant themselves to devote psychological resources to their own maintenance and development.

Phenomenologically, higher Self-Cathexis may be expressed through stable self-worth, realistic self-acceptance, and a robust sense of personal significance, whereas low Self-Cathexis may involve persistent self-doubt, habitual self-devaluation, and the tendency to minimize one’s own subjective importance (19, 22). Importantly, high Self-Cathexis is not equivalent to global self-esteem, nor does a higher level necessarily indicate adaptive functioning. Strong self-investment may take an integrated form characterized by secure self-confidence and self-acceptance, but it may also become rigid or defensive, manifesting as inflated self-importance, interpersonal entitlement, and pathological grandiose narcissistic functioning (22, 26, 45). Self-Cathexis therefore concerns the magnitude and organization of psychological investment in the self rather than the positive or negative evaluation of specific self-attributes.

The second core dimension, Self-Space Need, refers to the amount of relational, psychological, and developmental space required for the self to unfold, expand, and maintain coherence. This dimension derives primarily from object relations theory and self-psychology, particularly Kohut’s account of mirroring and idealizing selfobject needs (6, 7). Mirroring experiences provide recognition and affective affirmation, whereas idealizing experiences allow individuals to derive stability, strength, and self-value from admired figures and internalized ideals. Together, these relational functions provide an experiential field within which the self can develop and consolidate. Self-Space Need abstracts these functions into a broader structural dimension: the extent to which self-organization depends on relational and environmental conditions that permit recognition, self-expression, autonomy, support, and continued development (15–18).

Self-Space Need is therefore conceptually related to, but broader than, specific desires for mirroring, idealization, or interpersonal validation. It includes a dispositional requirement for social recognition, legitimate self-assertion, relational autonomy, and non-intrusive support. When adequate relational space is unavailable, individuals may experience self-contraction, frustration, heightened dependence, or vulnerability. Conversely, relational space that is insufficiently structured by internal boundaries may permit diffuse or poorly integrated self-expansion. Thus, a greater Self-Space Need is not inherently pathological; its psychological implications depend on the individual’s level and organization of Self-Cathexis, the adequacy of internal boundaries, and the responsiveness of the relational environment.

The bidimensional model therefore conceptualizes self-structure as the dynamic configuration of Self-Cathexis and Self-Space Need rather than as a single continuum extending from healthy to pathological narcissism (15, 16). At the measurement level, however, these theoretical dimensions may manifest through multiple related first-order facets, and empirical modeling should therefore evaluate whether a simple hierarchical structure adequately represents the complexity of self-structural organization. Within this framework, self-density is retained as a heuristic descriptor of the relative balance between the psychological investment allocated to the self and the relational space required for its maintenance. Relatively high self-density describes a more compact and bounded self-organization, in which substantial self-investment is maintained within comparatively limited relational space, whereas relatively low self-density describes a more diffuse organization in which available self-investment is insufficient relative to the relational and developmental space required by the self-system. Self-density is not treated as a third independent dimension or as a mathematically calculated ratio in the present research; rather, it provides a conceptual language for describing the relative concentration or diffuseness of self-organization within the two-dimensional space. Importantly, self-density is conceptualized as a theoretically informed heuristic construct rather than a directly quantifiable energy-density index, and it is not operationalized as a statistical ratio or numerical calculation in the present study.

This framework provides a structural account of both the relative stability and situational fluctuation of narcissistic phenotypes. Individuals with higher Self-Cathexis may display confident, agentic, and self-assured functioning when their relational conditions support self-expression and autonomy, but may shift toward rigid, defensive, or antagonistic functioning when the relational space required for self-maintenance is constrained or threatened (22, 26, 38). In contrast, individuals with relatively high Self-Space Need and low Self-Cathexis may depend more strongly on external recognition to preserve self-coherence and may become especially susceptible to shame, emptiness, insecurity, and interpersonal vulnerability when relational support is unavailable (21, 23, 46, 47). The bidimensional model does not replace existing trait models of grandiose and vulnerable narcissism. Instead, it offers a structural hypothesis for understanding why these phenotypes may coexist, alternate across situations, or emerge through heterogeneous developmental pathways (31, 33, 35, 48).

Crossing the two core continuous dimensions yields four theoretically interpretable self-structural configurations. First, relatively high Self-Cathexis combined with high Self-Space Need describes an expansive and agentic self-organization, which may support adaptive self-development but may also be associated with grandiose self-expansion when poorly regulated (22, 26, 38). Second, high Self-Space Need combined with low Self-Cathexis describes a vulnerable, void-type self-structure, marked by fragile self-worth, pervasive insecurity, and an urgent need for interpersonal recognition and relational holding (21, 23, 46). Third, relatively low levels of both dimensions describe a constricted configuration involving limited self-investment, reduced self-expansion, and diminished motivation for self-assertion or interpersonal differentiation (13, 47). Fourth, high Self-Cathexis combined with low Self-Space Need describes a compact, rigid, and antagonistic configuration, which may overlap with antagonistic or malignant narcissistic features but should not be equated with a clinical diagnosis (12, 39, 40, 49).

These configurations are not intended as categorical personality types or diagnostic classifications. Rather, they constitute a heuristic map of how different combinations of self-investment and relational space requirements may give rise to diverse patterns of narcissistic and broader self-functioning. This structural map also provides the theoretical rationale for the person-centered analyses used in the present research, which examine whether interpretable profiles emerge within the continuous SOSS measurement space.

## 2 Operationalizing the bidimensional model: the present research

Existing theoretical frameworks and psychometric measures have substantially advanced the empirical understanding of narcissistic traits, self-esteem regulation, and explicit self-representational content. Nevertheless, few instruments are designed to directly assess the structural organization through which psychological investment is allocated to the self and relational conditions are used to support self-development, coherence, and regulation. A psychometrically rigorous measure of self-structure may therefore complement existing trait-oriented approaches by shifting the focus from specific self-beliefs and observable narcissistic phenotypes to the broader organizational architecture underlying self-functioning.

To address this gap, the Scale of Self Structure (SOSS) was developed to operationalize the two theoretically proposed dimensions of Self-Cathexis and Self-Space Need in everyday psychological and interpersonal functioning (15, 16). Self-Cathexis denotes the degree of enduring psychological investment directed toward the self, whereas Self-Space Need reflects the amount of relational and developmental space individuals require to sustain self-expression, structural consolidation, and adaptive self-regulation. These dimensions are proposed to provide a parsimonious structural framework for examining individual differences in narcissistic functioning and broader patterns of self-organization (15, 16).

At the measurement level, the SOSS was designed to assess these theoretical dimensions through four theoretically specified first-order facets. These first-order facets are not intended to correspond directly to the four theoretical configurations described above. Rather, they provide multiple observable manifestations through which the two continuous structural dimensions can be assessed.

Positive Self represents an adaptive facet of Self-Cathexis, encompassing stable internal self-worth, realistic self-acceptance, and the capacity to recognize personal value without requiring inflated entitlement or interpersonal superiority (6, 19, 45). Self-Center represents a more agentic and self-prioritizing facet of Self-Cathexis, including self-centered cognition, expectations of social recognition, and the tendency to prioritize one’s own perspective, needs, and interests in interpersonal situations (20, 22, 36). Together, Positive Self and Self-Center capture two qualitatively different expressions of psychological investment in the self: one centered on internally grounded self-worth and the other on the interpersonal centrality and prioritization of the self.

Idealized Identification represents one facet of Self-Space Need and reflects the use of admired figures, authoritative models, and internalized ideals as sources of stability, direction, and self-value (6, 7, 50). This facet is theoretically related to Kohut’s idealizing selfobject function, through which connection with valued external or internalized figures may support self-development and structural consolidation. Vulnerable Self captures insecurity regarding self-worth, sensitivity to rejection and interpersonal neglect, and a heightened need for external relational support to preserve self-coherence (21, 23, 46, 47). Together, Idealized Identification and Vulnerable Self represent two distinct manifestations of Self-Space Need: the use of idealized relational or symbolic resources for self-development and the need for interpersonal recognition and support when self-coherence is vulnerable.

The correspondence between the two higher-order dimensions and the four first-order facets is therefore theoretical rather than mechanically deterministic. Positive Self and Self-Center are expected to load primarily on Self-Cathexis, whereas Idealized Identification and Vulnerable Self are expected to load primarily on Self-Space Need; however, the broader bidimensional model assumes that these facets function within an interconnected self-organizational system. The four first-order facets thus operationalize the two structural dimensions, whereas the four configurations described in the preceding section refer to person-level combinations of scores across those dimensions.

**Figure 1:**
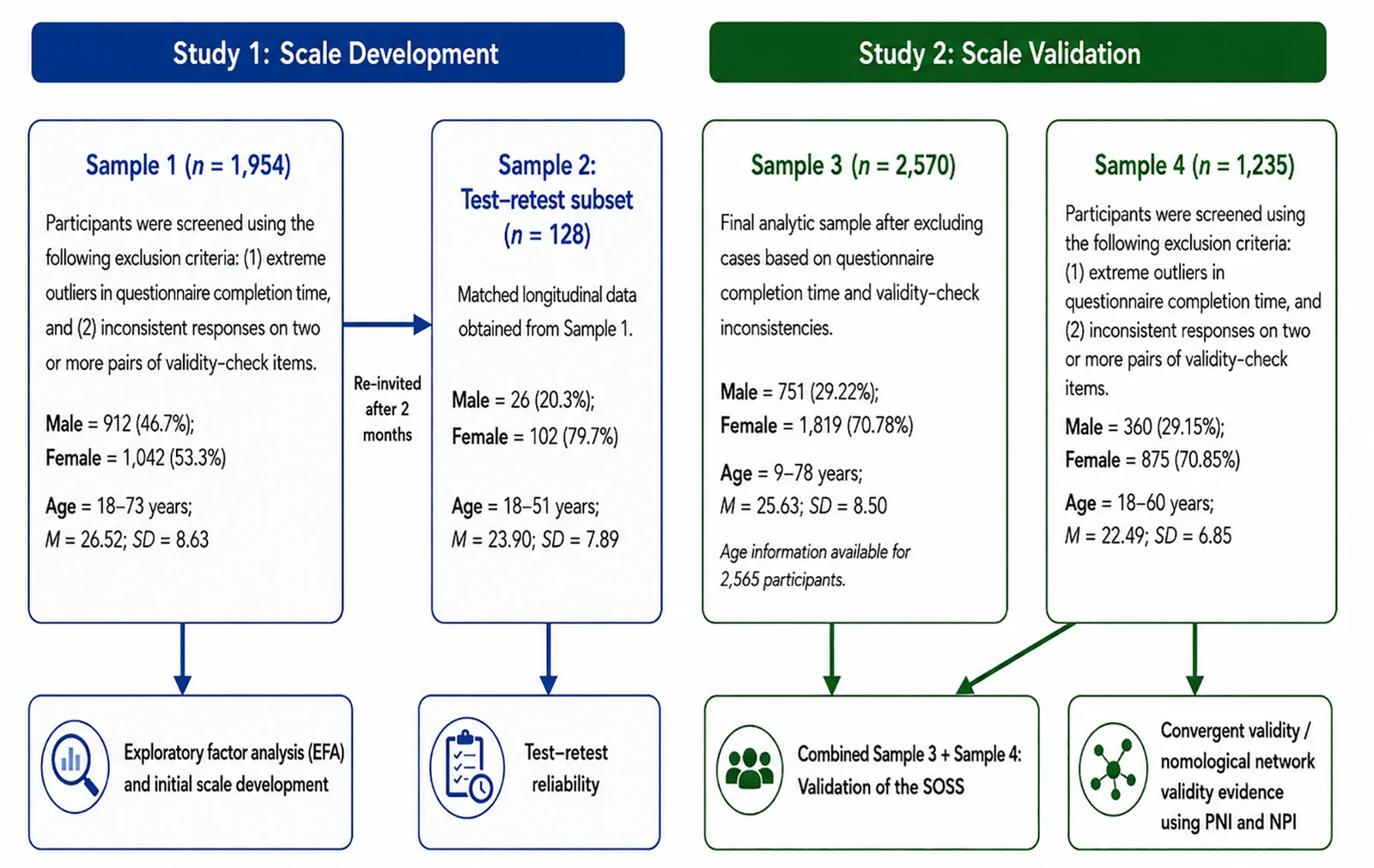
Flowchart of participant recruitment and data allocation. This flowchart illustrates participant recruitment and sample allocation across Study 1 and Study 2. Samples 3 and 4 were pooled for structural validation of the SOSS. Only participants in Sample 4 completed the NPI and PNI scales to obtain evidence for convergent and nomological network validity.

The present research comprised two empirical studies designed to develop and evaluate the SOSS. Study 1 involved initial item generation, iterative item refinement, exploratory factor analysis (EFA), internal consistency assessment, and examination of test–retest reliability. Study 2 examined the proposed factor structure using confirmatory factor analysis (CFA) and exploratory structural equation modeling (ESEM), and evaluated construct validity through discriminant validity, convergent and nomological associations with narcissism measures, and person-centered profile analysis. Across the two studies, the SOSS was expected to demonstrate a replicable multidimensional factor structure consistent with the proposed bidimensional theoretical framework, adequate internal consistency and temporal stability, theoretically coherent associations with established measures of grandiose and vulnerable narcissism, and interpretable person-centered profiles within the self-structure space. More specifically, Positive Self and Self-Center were expected to represent theoretically related facets of Self-Cathexis, whereas Idealized Identification and Vulnerable Self were expected to represent theoretically related facets of Self-Space Need. The factor structure was examined through competing measurement models to determine how these theoretically proposed dimensions were best represented empirically. Person-centered analyses were further expected to identify interpretable configurations based on the joint distribution of Self-Cathexis and Self-Space Need, without assuming that empirical profiles would necessarily reproduce all theoretically proposed configurations in exact categorical form. An overview of participant recruitment and data allocation across Study 1 and Study 2 is presented in **Figure 1**.

## 3 Study 1: development and reliability of SOSS

### 3.1 Methods

#### 3.1.1 Participants

Sample 1: Participants were screened using the following exclusion criteria: (1) extreme outliers in questionnaire completion time, and (2) inconsistent responses on two or more pairs of validity-check items. The final valid dataset comprised 1,954 participants (male: 912, 46.7%; female: 1,042, 53.3%), aged 18–73 years (M = 26.52, SD = 8.63). Educational backgrounds included: junior high school (n = 3, 0.2%), high school (n = 48, 2.5%), bachelor’s degree (n = 1,371, 88.6%), master’s degree (n = 154, 7.9%), doctoral degree (n = 16, 0.8%), and other (n = 2, 0.1%).

Sample 2: Two months after the initial administration to Sample 1, participants were re-invited to complete the scale. Matched longitudinal data were obtained from 128 participants (male: 26, 20.3%; female: 102, 79.7%), aged 18–51 years (M = 23.90, SD = 7.89).

#### 3.1.2 Research instruments

Demographic Information: Included basic variables such as gender, age, educational level, and only-child status.

Scale of Self Structure (SOSS): The preliminary version was developed by a research team consisting of 1 associate professor of clinical psychology (psychoanalyst, IPA-DM) and 5 clinical/health psychology graduate students with 2–3 years of clinical experience. Based on the dual-dimensional theory of self-structure and clinical practice, the team generated initial items through iterative discussions to evaluate content validity. After deleting ambiguous items and refining the scale structure, 37 items were retained: 17 items for the Self-Cathexis dimension and 20 items for the self-space need dimension. Respondents rated their agreement with each statement on a Likert 5-point scale ranging from 1 (completely disagree) to 5 (completely agree).

Validity-check Items: To exclude random responses, 6 items (three pairs) were embedded. Participants were required to correctly answer at least two pairs.

Social Desirability Items: To control for high social desirability bias, 24 items from the MCMI-IV (Millon Clinical Multiaxial Inventory IV) were included (51).

#### 3.1.3 Procedure

Data collection was conducted via WJX (Questionnaire Star) and a psychological assessment system. Participants received free access to an online course package upon questionnaire submission. Statistical analyses were performed using R 4.2.3, including descriptive statistics, item analysis, exploratory factor analysis (EFA), internal consistency reliability assessment, test-retest reliability analysis, and independent samples t-tests.

#### 3.1.4 Statistical analysis

All analyses were conducted in R 4.2.3. Preliminary item screening was conducted using social desirability assessment, item-total correlations, and extreme-group discrimination analyses. Items were evaluated based on their correlations with the total score and their ability to differentiate between participants with high and low total scores.

Exploratory factor analysis (EFA) was conducted to examine the latent structure of the preliminary SOSS items. Principal axis factoring with oblimin rotation was employed because the underlying factors were expected to be correlated. Factor retention was guided by scree plot inspection, eigenvalues, and theoretical interpretability. Alternative factor solutions were examined, and item retention was determined according to predefined criteria: items with factor loadings below.30 across all factors or items exhibiting substantial cross-loadings (difference between the two highest loadings <.20) were removed.

Internal consistency reliability of the retained SOSS items was evaluated using Cronbach’s alpha coefficients for the total scale and identified dimensions.

Temporal stability was examined using a two-month test–retest design. Intraclass correlation coefficients (ICC) were calculated using a two-way random-effects model with absolute agreement for single measurements [ICC(A,1)]. Pearson correlations between Time 1 and Time 2 scores were additionally calculated as supplementary indicators of rank-order consistency. Gender-stratified analyses were conducted to examine whether test–retest reliability differed between male and female participants. Differences between gender-specific ICC estimates were evaluated using bootstrap confidence interval comparisons, and Time × Gender interactions were examined to assess potential moderation effects.

Because only a subset of participants completed the retest assessment, attrition analyses were conducted to evaluate potential bias associated with differential retention. Participants who completed the follow-up assessment were compared with those who did not return on demographic characteristics and baseline SOSS scores. Logistic regression models were used to identify predictors of retest participation, and inverse probability weighting (IPW) sensitivity analyses were conducted to examine whether potential attrition bias influenced test–retest reliability estimates. Weighted ICC estimates were compared with the original ICC estimates to evaluate the robustness of temporal stability findings.

### 3.2 Results

#### 3.2.1 Preliminary item screening

Social desirability analysis on Sample 1 (n = 1,954) revealed that all items exhibited acceptable social desirability scores (< 0.6). Item-total correlations were significant (p <.01) for all items, with coefficients exceeding.30 except for items 19, 25, and 30. All items demonstrated significant differences (*p* < .001) between high- and low-scoring groups (27% extremes), indicating satisfactory item discrimination.

#### 3.2.2 Exploratory factor analysis

Exploratory factor analysis (EFA) was conducted on the 37-item preliminary version of the Scale of Self Structure (SOSS) using Sample 1 data (n = 1,954). The Kaiser-Meyer-Olkin (KMO) measure of sampling adequacy was.912, and Bartlett’s test of sphericity was significant (χ²(666) = 22,934.511, p <.001), confirming factorability.

**Figure 2:**
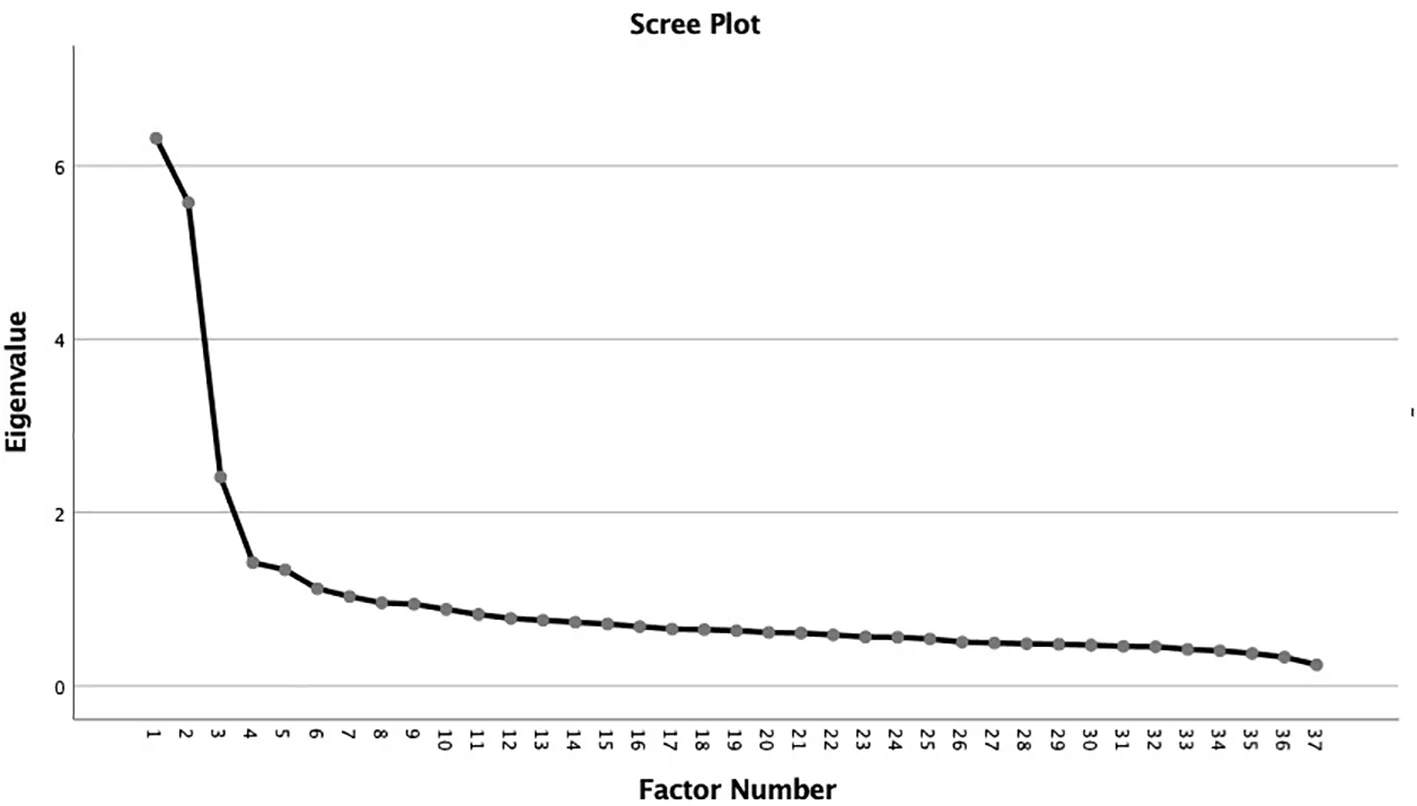
Scree plot of EFA for the preliminary SOSS. This scree plot presents eigenvalues of all extracted factors from exploratory factor analysis on the initial item pool. The inflection points on the curve guided the decision on the number of factors retained for follow-up psychometric analyses. EFA, exploratory factor analysis.

Principal axis factoring with oblimin rotation (allowing correlated factors) was applied without constraining the number of factors. Seven factors with eigenvalues greater than 1 emerged, cumulatively explaining 51.914% of the total variance. The scree plot is presented in **Figure 2**.

Factor extraction was determined by the inflection points in the scree plot. As shown in **Figure 2**, the curve exhibited inflection points at the 3rd and 5th factors, suggesting 2-factor or 4-factor solutions. Subsequent EFA models were tested with 2 and 4 factors, applying the following item deletion criteria.

The 2-factor EFA eliminated items 24, 32, and 36, explaining 33.910% of the cumulative variance. The 4-factor EFA removed items 5, 6, 19, 25, 29, 30, 35, 36, and 37, achieving a cumulative explained variance of 47.390%. The 4-factor solution was retained due to its superior interpretability and higher variance explanation. The final four-factor solution comprised 28 items and demonstrated a multidimensional structure consisting of four related factors (**Table 1**). The four first-order factors were labeled Positive Self, Self-Center, Idealized Identification, and Vulnerable Self. The four first-order factors were labeled Positive Self, Self-Center, Idealized Identification, and Vulnerable Self. The complete item-level loading matrix is provided in **Supplementary Table 1**. Conceptually, Positive Self and Self-Center were interpreted as reflecting self-investment-related processes, whereas Idealized Identification and Vulnerable Self were interpreted as reflecting relational and self-cohesion-related processes.

**Table 1:** Final factor structure and reliability of SOSS.

Higher-order dimension	First-order factor	Item no.	No. of items	Loading range	Cronbach’s α
Self-Cathexis	Positive Self	1, 2, 3, 4, 7, 8, 9, 11, 15	9	.362–.851	.836
	Self-Center	10, 12, 13, 14, 16, 17, 18	7	.404–.620	.777
Self-Space Need	Idealized Identification	20, 21, 22, 23, 24	5	.445–.650	.762
	Vulnerable Self	26, 27, 28, 31, 32, 33, 34	7	.380–.594	.733

Self-Cathexis and self-space need represent the two higher-order dimensions of the SOSS. Loading range indicates the minimum and maximum standardized factor loadings for retained items within each factor. Cronbach’s α coefficients are reported for each first-order factor. Full item-level factor loadings are presented in **Supplementary Table 1**.

#### 3.2.3 Internal consistency reliability

Analysis of internal consistency using Sample 1 data (n = 1,954) indicated satisfactory reliability for the final 28-item SOSS. The total scale yielded a Cronbach’s α of.816. The theoretically derived composite dimensions also demonstrated acceptable internal consistency, with α coefficients of.789 for Self-Cathexis and.809 for Self-Space Need. Reliability coefficients for the four first-order factors are summarized in **Table 1**.

#### 3.2.4 Test-retest reliability

Test–retest reliability was assessed in a retest subsample (n = 128) over a two-month interval using two-way absolute-agreement, single-measure intraclass correlation coefficients, ICC(A,1). The SOSS total score demonstrated good test–retest reliability, ICC(A,1) = .870, 95% CI [.819,.907]. The two theoretically derived composite dimensions also showed acceptable-to-good reliability: Self-Cathexis, ICC(A,1) = .794, 95% CI [.698,.858], and Self-Space Need, ICC(A,1) = .799, 95% CI [.725,.854]. ICC estimates for the four first-order factors ranged from.668 to.776. Pearson test–retest correlations, provided as supplementary indices of rank-order consistency, were.874 for the total score and ranged from.675 to.811 across the four first-order factors and two theoretically derived composite dimensions. Complete results are presented in **Supplementary Table 2**.

Gender-stratified analyses indicated good-to-excellent test–retest reliability for the total score among both male participants (n = 28), ICC(A,1) = .919, 95% CI [.834,.961], and female participants (n = 100), ICC(A,1) = .843, 95% CI [.775,.892]. The direct bootstrap comparison of the two ICC estimates was not significant, ΔICC*_female-male_* = −.076, 95% CI [−.178,.050], p = .188. The Time × Gender interaction was also not significant, p = .967. Across the total score and all SOSS dimensions, none of the gender differences in ICC estimates remained significant after correction for multiple comparisons. Detailed gender-stratified results are provided in **Supplementary Table 3**.

Attrition analyses were further conducted to evaluate whether differential retention affected the test–retest reliability estimates. Compared with participants who did not complete the follow-up assessment, retest completers were younger and differed in gender distribution; however, baseline SOSS total scores did not significantly differ between retained and attrited participants. Although some differences were observed for specific first-order factors, logistic regression analyses indicated that baseline SOSS factors did not significantly predict retest participation. Furthermore, inverse probability weighting (IPW)-adjusted ICC estimates were highly consistent with the original ICC estimates, suggesting that potential attrition bias had minimal influence on the temporal stability estimates of the SOSS (**Supplementary Table 4**).

## 4 Study 2: validity of SOSS

### 4.1 Methods

#### 4.1.1 Participants

Sample 3: The final analytic sample comprised 2,570 participants after excluding cases based on questionnaire completion time and validity-check inconsistencies. The sample included 751 males (29.22%) and 1,819 females (70.78%). Age information was available for 2,565 participants, with ages ranging from 9 to 78 years (M = 25.63, SD = 8.50).

Sample 4: A total of 1,235 participants were included in the final valid dataset (male: 360, 29.15%; female: 875, 70.85%), aged 18–60 years (M = 22.49, SD = 6.85), after applying the same exclusion criteria based on questionnaire completion time and validity-check inconsistencies. In addition to completing the SOSS, this sample also completed the Narcissistic Personality Inventory (NPI) and the Pathological Narcissism Inventory (PNI) for convergent and nomological validity analyses.

#### 4.1.2 Instruments

Scale of Self Structure (SOSS): The 28-item scale developed in Study 1 was used to assess self-structure, comprising two dimensions (*Self-Cathexis* and *self-space need*) and four factors (*Positive Self, Self-Center, Idealized Identification*, and *Vulnerable Self*). Items were rated on a Likert 5-point scale from 1 (completely disagree) to 5 (completely agree).

13-Item Narcissistic Personality Inventory (NPI-13): The Chinese version employs a forced-choice format to measure grandiose narcissism, with 13 items across three dimensions: *Leadership/Authority*, *Grandiose Self-Exhibition*, and *Entitlement/Exploitativeness* (52).

Pathological Narcissism Inventory (PNI): The Chinese adaptation, revised from Pincus et al. and Wright et al., assessed vulnerable narcissism using a 34-item subset from the second-order factor model. Responses were recorded on a 6-point scale (1 = not at all like me to 6 = very much like me) (23, 44, 53).

MCMI-IV: The narcissistic personality disorder subscale of MCMI-IV (51), revised by the Personality Organization Assessment Laboratory at Peking University’s School of Psychological and Cognitive Sciences, included 16 dichotomous items (0/1 scoring).

Validity-check Items: Six paired validity-check items were embedded; participants were required to correctly answer at least two pairs to ensure data validity.

#### 4.1.3 Procedure

Data collection utilized WJX (Questionnaire Star) and a psychological assessment system. Participants received personalized feedback and complimentary access to an online course upon completion.

#### 4.1.4 Statistical analysis

Analyses were conducted in R 4.2.3, including descriptive statistics, internal consistency reliability assessment, confirmatory factor analysis (CFA), exploratory structural equation modeling (ESEM), measurement invariance analysis, discriminant validity analysis, convergent and nomological validity evaluation, and cluster analysis.

Given that SOSS items were assessed using five-point Likert scales, factor analyses treated item responses as ordinal indicators and were estimated using the weighted least squares mean and variance adjusted estimator (WLSMV). To evaluate the latent structure of the SOSS, a series of competing measurement models were examined, including a two-factor model reflecting the proposed bidimensional conceptual framework (Self-Cathexis and Self-Space Need), a four-factor model representing the theoretically specified first-order facets (Positive Self, Self-Center, Idealized Identification, and Vulnerable Self), theoretically derived higher-order models, and a four-factor exploratory structural equation model (ESEM).

The higher-order models were evaluated as theoretically motivated alternative representations of the SOSS structure rather than being assumed as the only possible measurement solution. A four-factor ESEM model was additionally examined because the theoretical conceptualization of self-structure allows for potential overlap among related self-organizational processes. Unlike traditional CFA models, ESEM permits theoretically meaningful cross-loadings while maintaining an interpretable factor structure.

Model fit was evaluated using robust comparative fit index (CFI), robust Tucker–Lewis index (TLI), root mean square error of approximation (RMSEA), and standardized root mean square residual (SRMR). Robust CFI and TLI values were reported because they provide more conservative estimates when ordinal indicators and non-normal response distributions are present.

As a sensitivity analysis, CFA models were additionally estimated by treating Likert-scale items as continuous indicators. These analyses were conducted to examine whether the overall conclusions regarding the latent structure were influenced by the treatment of ordinal response categories. In addition, measurement invariance analyses were conducted to examine whether the retained four-factor ESEM structure was comparable across gender groups. Configural, metric, and scalar invariance models were sequentially evaluated using WLSMV estimation, and invariance decisions were based on changes in robust fit indices, including ΔCFI, ΔRMSEA, and ΔSRMR.

Discriminant validity was assessed using the Fornell–Larcker criterion based on the four-factor CFA solution. Average variance extracted (AVE) values were calculated from standardized factor loadings of the four-factor CFA model, and the square roots of AVE were compared with inter-factor correlations to evaluate whether each factor shared greater variance with its own indicators than with other factors.

Internal consistency was evaluated using both Cronbach’s alpha and McDonald’s omega coefficients. Cronbach’s alpha was calculated as a traditional estimate of internal consistency, whereas McDonald’s omega was additionally estimated to provide a model-based reliability assessment. Omega coefficients and their 95% confidence intervals were estimated using bootstrap procedures based on the retained four-factor measurement structure. Reliability was examined for the total SOSS score, the two theoretically derived dimensions (Self-Cathexis and Self-Space Need), and the four first-order facets (Positive Self, Self-Center, Idealized Identification, and Vulnerable Self). Given that Study 2 served as an independent validation study of the factor structure derived from Study 1, temporal stability was not assessed in this sample; test–retest reliability was examined in Study 1 using a separate retest sample.

To evaluate the robustness of the exploratory clustering structure, we additionally conducted a split-half stability check using the existing dataset. Approximately 50% of participants were randomly selected, and the clustering procedure was repeated under the same preprocessing and analytic specifications. The resulting subsample solution was visualized via a scatter plot for direct comparison with the full-sample clustering pattern.

### 4.2 Results

#### 4.2.1 Factor structure validation

**Figure 3:**
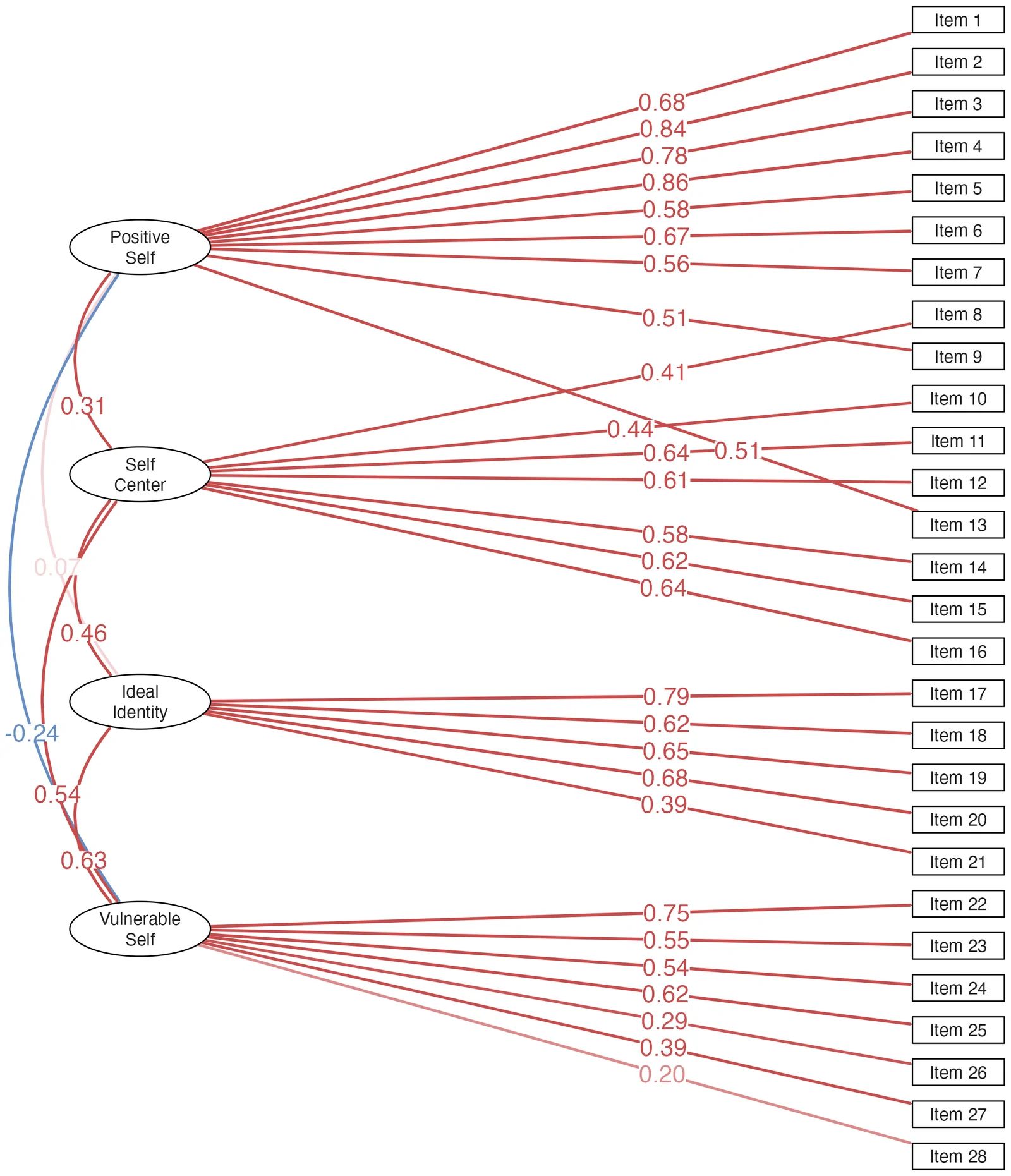
Four-factor CFA model of the SOSS. This image displays standardized factor loadings of four latent facets (Positive Self, Self-Center, Idealized Identification, Vulnerable Self) from confirmatory factor analysis of the preliminary SOSS. CFA, confirmatory factor analysis.

Using combined data from Samples 3 and 4 (n = 3,805), confirmatory factor analyses were conducted to examine whether the factor structure identified in Study 1 could be replicated in an independent sample. Because SOSS items were treated as ordinal indicators, all models were estimated using the WLSMV estimator with robust fit indices. The four-factor CFA model representing Positive Self, Self-Center, Idealized Identification, and Vulnerable Self showed improved fit compared with the simpler two-factor solution; however, model fit remained suboptimal under the stricter estimation approach (**Table 2**; **Figure 3**). Therefore, additional theoretically motivated measurement models, including higher-order CFA models and a four-factor ESEM model, were examined to identify the most appropriate representation of the SOSS structure.

**Table 2:** Fit indices of competing measurement models for the SOSS in Study 2.

Model	χ² (scaled)	df	Robust CFI	Robust TLI	Robust RMSEA (90% CI)	SRMR
2-factor CFA	21913.579	349	0.589	0.555	.106 [.104,.108]	0.127
4-factor CFA	9251.604	344	0.783	0.761	.078 [.076,.080]	0.079
Higher-order Model A	—	—	—	—	—	—
Higher-order Model B	12331.059	346	0.762	0.741	.081 [.079,.083]	0.095
Higher-order Model C	—	344	—	—	—	—
4-factor ESEM	3580.329	272	0.901	0.862	.059 [.057,.061]	0.032

Model fit indices were estimated using the weighted least squares mean and variance adjusted (WLSMV) estimator with ordinal indicators. Robust comparative fit index (CFI) and robust Tucker–Lewis index (TLI) are reported. The root mean square error of approximation (RMSEA) is presented with its 90% confidence interval (CI). SRMR = standardized root mean square residual. Higher-order Model A failed to converge under the WLSMV estimation procedure; therefore, fit indices were unavailable. Higher-order Model C converged but did not yield robust fit indices, and therefore could not be meaningfully evaluated. The four-factor ESEM model demonstrated the best relative fit among the tested measurement models.

To examine whether the proposed bidimensional theoretical framework could be represented as a hierarchical measurement structure, theoretically specified higher-order CFA models were evaluated as alternative models. Model A specified Self-Cathexis as encompassing Positive Self and Self-Center, whereas Self-Space Need comprised Idealized Identification and Vulnerable Self. Model B represented an alternative configuration in which Positive Self was assigned to Self-Cathexis, while Self-Center, Idealized Identification, and Vulnerable Self loaded onto Self-Space Need. Model C allowed Self-Center to contribute to both higher-order dimensions. Under the stricter WLSMV estimation approach, Model A failed to converge, and Model C did not yield robust fit indices, preventing meaningful evaluation of these two hierarchical models. Model B converged but showed inadequate fit (robust CFI = .762, robust TLI = .741, robust RMSEA = .081, SRMR = .095), suggesting that the hypothesized hierarchical representation did not provide an adequate measurement model for the SOSS (**Table 2**).

**Figure 4:**
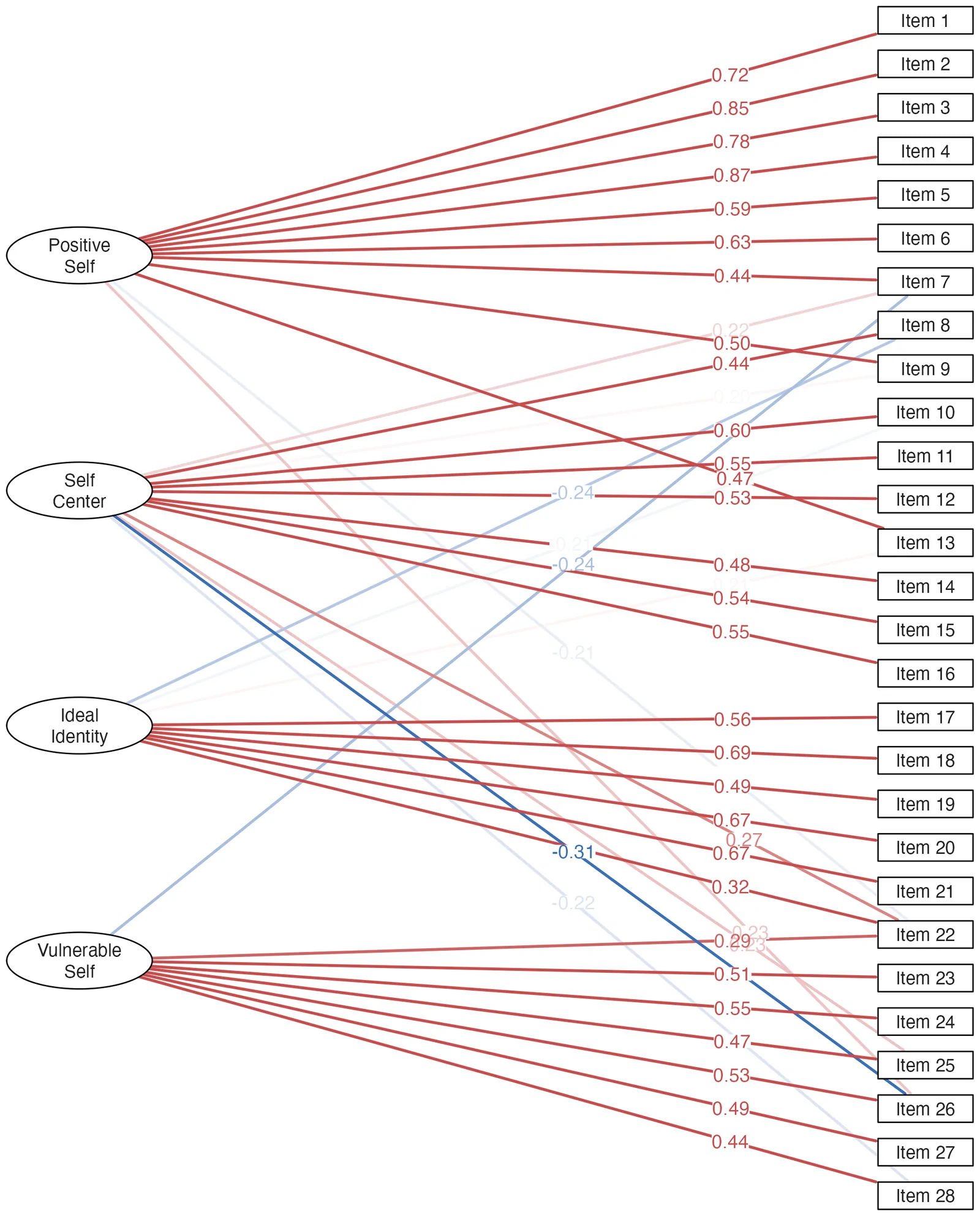
Four-factor ESEM model of the SOSS. This image reports standardized item loadings from the four-factor exploratory structural equation modeling solution. This model permits theoretically justified cross-loadings while preserving the four latent facets: Positive Self, Self-Center, Idealized Identification, and Vulnerable Self. ESEM, exploratory structural equation modeling.

Given the possibility that items may reflect overlapping aspects of self-structural organization, a four-factor ESEM model was further examined. The ESEM model allowed theoretically meaningful cross-loadings while maintaining the four-factor conceptual structure. Among all tested models, the four-factor ESEM model demonstrated the best relative fit (robust CFI = .901, robust TLI = .862, robust RMSEA = .059, 90% CI [.057,.061], SRMR = .032), providing the most adequate empirical representation of the SOSS structure (**Table 2**, **Figure 4**). The item-level standardized loading pattern from the target-rotated ESEM solution, including primary loadings and theoretically meaningful cross-loadings, is presented in **Supplementary Figure 1**. Sensitivity analyses using continuous-variable CFA produced a comparable pattern of model comparison and are presented in **Supplementary Table 5**.

Additional measurement invariance analyses were conducted to examine whether the four-factor ESEM structure was comparable across gender groups. Configural, metric, and scalar invariance were supported based on changes in robust fit indices, suggesting that the factor structure, factor loadings, and item thresholds were comparable across males and females. Detailed invariance results are presented in **Supplementary Table 6**.

#### 4.2.2 Internal consistency reliability

Internal consistency reliability was examined in the combined validation sample (Samples 3 and 4; n = 3,805) using both Cronbach’s alpha and McDonald’s omega coefficients. The total SOSS demonstrated satisfactory internal consistency (α = .795, ω = .859, 95% CI [.849,.868]). At the dimensional level, Self-Cathexis showed good reliability (α = .811, ω = .846, 95% CI [.845,.864]), whereas Self-Space Need demonstrated acceptable reliability (α = .744, ω = .727, 95% CI [.723,.779]). Across the four first-order facets, reliability coefficients ranged from acceptable to moderate (α = .657–.841; ω = .592–.821), with Positive Self showing the highest internal consistency and Vulnerable Self showing comparatively lower reliability. Complete reliability coefficients are presented in **Supplementary Table 7**.

#### 4.2.3 Discriminant validity

Discriminant validity of the SOSS was examined using combined data from Samples 3 and 4 (n = 3,805). Given that the unsupported hierarchical models did not provide an adequate representation of the data, discriminant validity was evaluated among the four theoretically specified first-order factors: Positive Self, Self-Center, Idealized Identification, and Vulnerable Self. Correlations among the four factors were generally low to moderate in magnitude, indicating that these facets represented related but distinguishable aspects of self-structure (**Supplementary Table 8**). In addition, the Fornell–Larcker criterion was examined using factor correlations and average variance extracted (AVE) estimates derived from the four-factor CFA model. The square roots of AVE were greater than the corresponding inter-factor correlations for all four factors, suggesting that each factor shared greater variance with its own indicators than with other latent factors (**Supplementary Table 9**). Together, these findings provided support for the discriminant validity of the four-factor SOSS structure.

#### 4.2.4 Convergent and nomological validity

To examine convergent and nomological validity, Pearson correlation analyses were conducted using Sample 4 (n = 1,235) to examine associations between SOSS scores and established measures of narcissism. The narcissism measures demonstrated acceptable internal consistency in the present sample, with the NPI showing α = .658 and ω = .673, and the PNI showing α = .951 and ω = .953.

At the theoretically derived dimensional level, Self-Cathexis and Self-Space Need scores were calculated by aggregating their corresponding first-order facet scores. Self-Cathexis showed a weak positive correlation with NPI scores (r = .180, p <.001) and a negligible association with PNI scores (r = −.042, p = .14), whereas Self-Space Need demonstrated a small positive correlation with NPI scores (r = .143, p <.001) and a strong positive correlation with PNI scores (r = .644, p <.001).

At the first-order factor level, Positive Self showed no significant association with NPI scores (r = .018, p = .53) but was negatively associated with PNI scores (r = −.341, p <.001). Self-Center and Idealized Identification showed positive associations with both narcissism measures, whereas Vulnerable Self demonstrated a strong positive association with PNI scores (r = .622, p <.001). Detailed correlation coefficients are presented in **Table 3**.

**Table 3:** Associations between SOSS dimensions/facets and narcissism measures.

SOSS score	NPI	PNI
Theoretically derived dimensions		
Self-Cathexis	.180***	-0.042
Self-space need	.143***	.644***
First-order factors		
Positive Self	0.018	-.341***
Self-Center	.329***	.346***
Idealized Identification	.205***	.454***
Vulnerable Self	.059*	.622***

*p<.05, **p<.01, ***p<.001. N = 1,235. SOSS, Scale of Self Structure; NPI, Narcissistic Personality Inventory; PNI, Pathological Narcissism Inventory. Self-Cathexis was calculated by aggregating Positive Self and Self-Center scores, whereas Self-Space Need was calculated by aggregating Idealized Identification and Vulnerable Self scores. Values represent Pearson correlation coefficients. Internal consistency of the narcissism measures in the present sample was α = .658 and ω = .673 for the NPI, and α = .951 and ω = .953 for the PNI.

Together, these findings provided evidence for the convergent and nomological validity of the SOSS by demonstrating theoretically meaningful associations between distinct aspects of self-structure and theoretically related narcissistic traits.

#### 4.2.5 Cluster analysis

**Figure 5:**
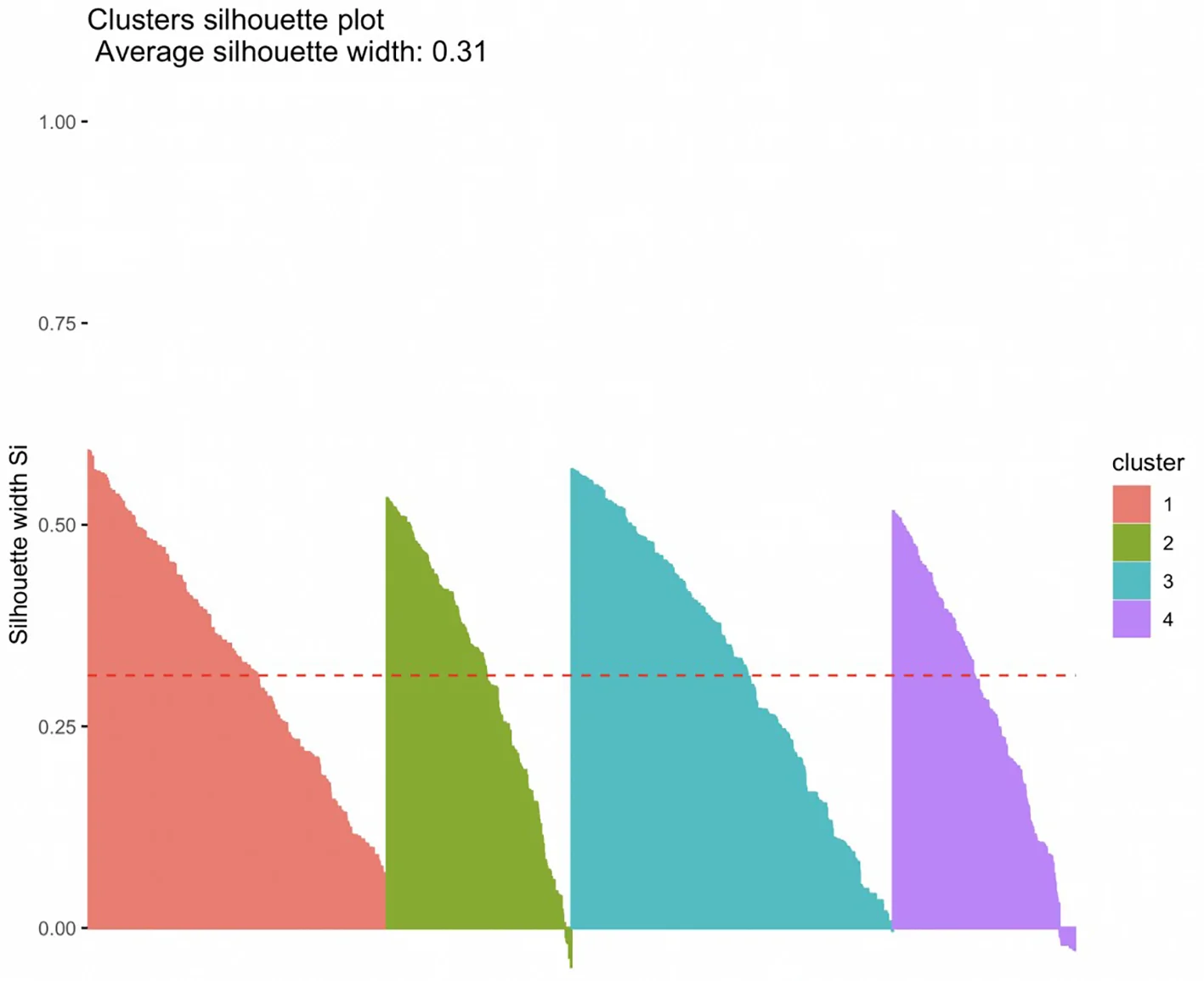
Silhouette plot of the four-cluster SOSS solution. This silhouette plot visualizes cluster discrimination for the four-cluster solution based on SOSS composite scores. The overall average silhouette coefficient equals 0.31, which signals weak-to-moderate differentiation between exploratory self-structure profiles. The horizontal dashed line marks the mean silhouette width across all participants.

A two-step cluster analysis was conducted using the combined data from Samples 3 and 4 (n = 3,805) to explore whether participants could be empirically grouped within the two-dimensional SOSS space defined by theoretically derived Self-Cathexis and Self-Space Need composite scores. The four-cluster solution yielded an average silhouette coefficient of.31, suggesting moderate but interpretable cluster separation. The silhouette plot for the four-cluster solution is presented in **Figure 5**.

Subsequent K-means clustering was conducted to characterize the four-cluster solution within the two-dimensional SOSS space. Cluster centers showed clear differentiation along Self-Cathexis and Self-Space Need, accounting for 61.6% of the between-cluster variance (**Table 4**). Follow-up ANOVAs indicated that the four clusters differed significantly on both dimensions, including Self-Cathexis (F(3, 3801) = 1808, p <.001) and Self-Space Need (F(3, 3801) = 2289, p <.001). These findings suggest that the identified clusters were primarily organized according to the theoretically derived two-dimensional framework. Detailed ANOVA results are provided in **Supplementary Table 10**.

**Table 4:** Cluster centers for the four-cluster solution of the SOSS.

Dimension (z score)	Cluster 1 (*n* = 1152)	Cluster 2 (*n* = 711)	Cluster 3 (*n* = 1238)	Cluster 4 (*n* = 704)	F
Self-Cathexis	0.54	1.11	-0.77	-0.65	1808
Self-Space Need	-0.40	1.04	0.51	-1.29	2289

SOSS, Scale of Self Structure. Cluster centers represent standardized composite scores on the two theoretically derived SOSS dimensions (Self-Cathexis and Self-Space Need). Positive values indicate scores above the sample mean, whereas negative values indicate scores below the sample mean. F values are from one-way analyses of variance comparing cluster differences on each dimension.

**Figure 6:**
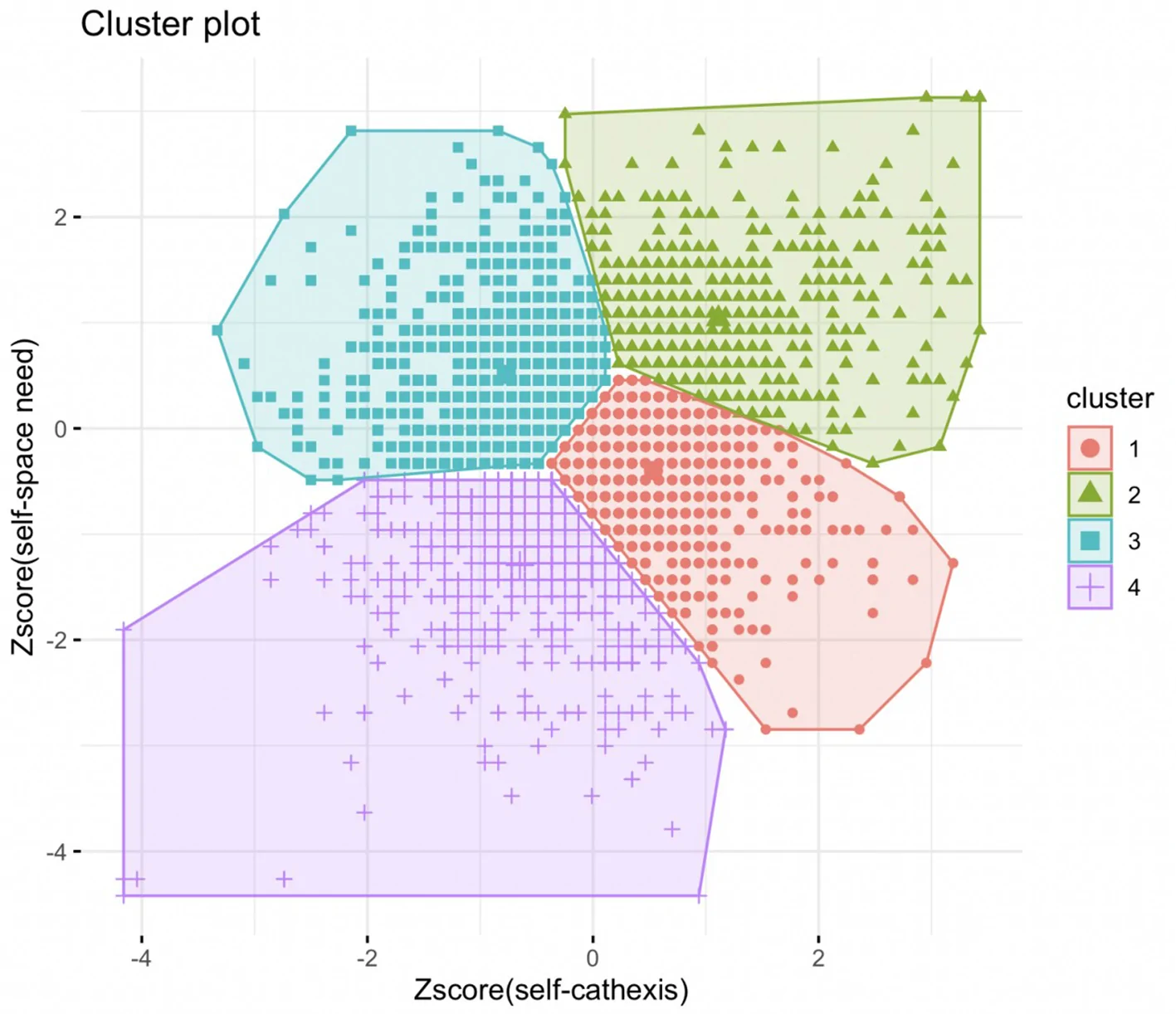
Four-cluster distribution in the two-dimensional SOSS space. This scatter plot displays the distribution of four empirical clusters within the bidimensional self-structure framework. The horizontal x-axis corresponds to standardized Self-Cathexis composite scores, and the vertical y-axis corresponds to standardized Self-Space Need composite scores. Distinct markers and shaded areas distinguish the four exploratory self-structure profiles. These clusters are interpreted as continuous dimensional configurations instead of discrete personality categories.

The clusters broadly corresponded to the four empirically derived regions of the two-dimensional space (**Figure 6**). The clusters broadly corresponded to the four theoretically derived regions of the two-dimensional space. Cluster 2 was characterized by relatively high levels of both Self-Cathexis and Self-Space Need, Cluster 3 by relatively low Self-Cathexis and high Self-Space Need, Cluster 4 by relatively low levels of both dimensions, and Cluster 1 by relatively high Self-Cathexis and relatively low Self-Space Need. A split-half scatterplot showed a similar distributional pattern, providing descriptive support for the robustness of the exploratory clustering solution, although cluster separation remained moderate (**Supplementary Figure 2**).

## 5 Discussion

### 5.1 The empirical organization of self-structure: from a bidimensional theoretical framework to a multidimensional measurement model

The present research developed and validated the SOSS as a quantitative measure of self-structural organization grounded in a bidimensional theoretical framework. Across two studies, the findings indicated that self-structure can be empirically characterized by four related but distinguishable components: Positive Self, Self-Center, Idealized Identification, and Vulnerable Self. Although the SOSS was initially developed based on the theoretical distinction between Self-Cathexis and Self-Space Need, the validation analyses suggested that these broad theoretical dimensions are expressed through multiple interconnected self-structural processes rather than a strictly hierarchical measurement structure.

The empirical findings suggest that self-structure is best understood as a multidimensional organization consisting of several related but distinguishable psychological processes. The four-factor structure identified in the present study captures encompassing Positive Self, Self-Center, Idealized Identification, and Vulnerable Self. These facets capture complementary aspects of self-organization, ranging from self-directed investment and self-focused regulation to reliance on idealized relational experiences and vulnerability in maintaining self-cohesion. Such a multidimensional representation is consistent with contemporary perspectives that conceptualize narcissistic functioning and self-related processes as dynamic configurations of interconnected psychological components rather than unitary traits (26, 32).

Importantly, the validation analyses further clarified that the empirical organization of self-structure may not be adequately represented by a strict hierarchical structure alone. Although Self-Cathexis and Self-Space Need provided the theoretical foundation for developing the SOSS, the hypothesized higher-order representation showed limited empirical support under the more stringent estimation approach. Instead, the four-factor ESEM solution provided a more adequate representation of the data by allowing theoretically meaningful overlap among related self-structural processes. This pattern suggests that the organization of the self may involve partially interconnected systems, in which different aspects of self-investment, relational experience, and self-regulation interact rather than operate as entirely separable hierarchical components.

Such a pattern is theoretically consistent with psychodynamic perspectives, which conceptualize the self as an evolving organization of representations, investments, and relational experiences rather than as a collection of independent psychological components. Classical psychoanalytic theories emphasized the role of self-directed investment and object-related experiences in shaping self-development, while self-psychological and object-relational approaches further highlighted the importance of relational contexts in maintaining self-cohesion and psychological continuity (3, 4, 6, 7, 17). From this perspective, the advantage of the ESEM solution may lie in its ability to capture the interconnected nature of self-related processes while preserving theoretically interpretable dimensions.

Additional analyses further indicated that the four-factor SOSS structure demonstrated measurement invariance across gender groups, providing preliminary evidence that the identified facets can be interpreted comparably across males and females. Taken together, these findings suggest that Self-Cathexis and Self-Space Need may be better understood as broad theoretical organizing principles rather than strictly hierarchical latent factors. The four empirically derived facets provide a more differentiated description of how self-investment, relational needs, and self-cohesion processes are organized across individuals. By integrating psychodynamic concepts with contemporary psychometric methodology, the SOSS offers a preliminary framework for examining self-structure as a multidimensional psychological system.

### 5.2 Self-cathexis and self-space need as theoretical dimensions of self-structure

Although the empirical structure of the SOSS was best represented by four related facets rather than a strict hierarchical model, the theoretical distinction between Self-Cathexis and Self-Space Need remains useful for interpreting the organization of these facets. These two concepts were not intended to represent independent diagnostic categories, but rather complementary aspects of self-organization: the psychological investment directed toward the self and the relational-developmental space required for maintaining and expanding self-experience.

The concept of Self-Cathexis is rooted in classical psychoanalytic theories of narcissism and self-development. Freud conceptualized narcissism through the allocation of libido toward the self, emphasizing self-directed investment as a fundamental aspect of narcissistic functioning (3). Later developments in ego psychology further differentiated self-related processes from broader ego functions, highlighting the role of self-representation and internal regulation in personality organization (4, 5). Within the SOSS framework, Self-Cathexis provides a conceptual basis for understanding facets related to positive self-investment and self-focused regulation, particularly Positive Self and Self-Center.

In contrast, Self-Space Need extends psychodynamic perspectives by emphasizing that self-development occurs within a relational and symbolic environment that provides opportunities for recognition, idealization, and self-cohesion. Kohut’s self psychology highlighted the importance of selfobject experiences, including mirroring and idealizing relationships, in supporting the development and maintenance of the self (6, 7). Similarly, object-relational theories have emphasized that internalized relational experiences contribute to the organization and continuity of self-experience (17, 18). In the SOSS framework, Self-Space Need provides a theoretical lens for understanding processes related to idealized relational experiences and vulnerability in maintaining self-cohesion, reflected in Idealized Identification and Vulnerable Self.

Importantly, the present findings suggest that these two theoretical dimensions should not be interpreted as simple oppositional poles or directly corresponding to healthy versus pathological forms of self-functioning. Rather, they represent broad organizing principles through which diverse self-structural processes may be understood. The four-factor ESEM solution indicates that these processes are partially interconnected, suggesting that self-organization emerges from the dynamic interplay between self-investment, relational experience, and self-regulation. Thus, Self-Cathexis and Self-Space Need may serve as conceptual coordinates for interpreting patterns of self-organization without imposing a rigid hierarchical structure on the underlying psychological processes.

### 5.3 Self-structural correlates of grandiose and vulnerable narcissistic traits

Contemporary narcissism research has increasingly emphasized that narcissistic functioning cannot be fully captured by a single trait dimension. Although grandiose and vulnerable narcissism are often conceptualized as distinct phenotypic expressions, accumulating evidence suggests that they reflect heterogeneous manifestations of underlying self-regulatory processes, including differences in self-esteem regulation, interpersonal orientation, and sensitivity to social evaluation (23, 26, 32).

Within this framework, the SOSS provides a complementary perspective by examining the self-structural processes that may underlie different narcissistic expressions. In the present study, Self-Cathexis showed a small positive association with grandiose narcissistic traits assessed by the NPI (r = .180, p <.001), whereas Self-Space Need demonstrated a substantially stronger association with pathological narcissistic traits assessed by the PNI (r = .644, p <.001). These findings suggest that different forms of narcissistic functioning may be differentially related to distinct aspects of self-organization rather than representing variations along a single continuum of self-investment.

The association between Self-Cathexis and NPI scores is theoretically consistent with the view that grandiose narcissistic traits involve elevated self-focused investment, self-enhancement, and regulation of self-esteem through positive self-evaluation (32, 54). However, the relatively modest magnitude of this association suggests that grandiose narcissistic traits cannot be reduced to self-investment alone. Instead, they may reflect a broader configuration involving interpersonal strategies, motivational orientations, and self-regulatory mechanisms. Moreover, the relatively moderate internal consistency of the NPI in the present sample (α = .658, ω = .673) should be considered when interpreting the magnitude of associations involving this measure.

In contrast, the stronger association between Self-Space Need and PNI scores may suggest that relational and self-cohesive processes are particularly relevant to vulnerable narcissistic features. Previous theoretical and empirical work has highlighted that vulnerable narcissistic traits are characterized by fragile self-esteem, hypersensitivity to evaluation, and dependence on external validation (23, 55). Consistent with this perspective, the present findings suggest that dimensions involving relational needs and self-cohesion may be particularly relevant for understanding individual differences in vulnerable narcissistic features.

At the facet level, Vulnerable Self showed the strongest association with PNI scores (r = .622, p <.001), further highlighting the relevance of self-cohesion-related processes in vulnerable narcissistic traits. Notably, Positive Self demonstrated a negative association with PNI scores (r = −.341, p <.001), suggesting that positive self-regard may represent a distinct aspect of self-organization rather than simply the opposite pole of vulnerability. Nevertheless, these findings should be interpreted as associations with narcissistic characteristics in a nonclinical population rather than evidence that the SOSS identifies narcissistic pathology or personality disorder. Future studies incorporating clinical samples, diagnostic assessments, and multimethod approaches are needed to determine whether SOSS dimensions provide additional information regarding personality functioning, narcissistic pathology, or related clinical phenomena.

### 5.4 Bridging psychodynamic theories of the self with contemporary psychometric approaches

A central contribution of the present study lies in the attempt to translate psychodynamic concepts of self-organization into measurable psychological constructs. Historically, psychoanalytic theories have provided rich conceptual accounts of how self-experience develops through processes of self-investment, internalization of relational experiences, and regulation of self-cohesion. However, many of these constructs have traditionally been examined primarily through clinical interpretation and theoretical formulation, making systematic empirical investigation challenging (3, 6, 20).

The development of the SOSS represents an effort to establish a quantitative framework for examining these theoretically derived aspects of self-organization. Rather than treating self-related processes as isolated personality traits, the SOSS conceptualizes self-structure as an organization of multiple interacting components involving self-investment, self-regulation, relational experience, and self-cohesion. This approach is consistent with contemporary perspectives emphasizing that personality functioning emerges from dynamic interactions among motivational, cognitive, affective, and interpersonal systems rather than from independent trait dimensions alone (32, 56).

Importantly, the present findings also highlight the value of allowing empirical models to refine theoretical assumptions. Although Self-Cathexis and Self-Space Need provided the conceptual foundation for scale development, the validation results indicated that self-structural processes were better represented by a multidimensional ESEM solution rather than a strict hierarchical model. This pattern suggests that psychodynamic constructs may benefit from measurement approaches capable of capturing complexity, overlap, and interdependence among psychological processes. Accordingly, the SOSS should not be interpreted as a direct operationalization of any single psychoanalytic theory, but rather as an empirically testable framework inspired by psychodynamic conceptualizations of the self. At the same time, the implications of the SOSS should be interpreted within the scope of the current empirical evidence. The present studies were conducted primarily in young, nonclinical Chinese samples, and no individuals with clinically diagnosed narcissistic personality disorder (NPD) were included. Therefore, the current findings do not establish the SOSS as a diagnostic instrument for NPD or other personality disorders, nor do they provide direct evidence for clinical decision-making or treatment planning. Instead, the present results provide preliminary evidence that self-structural dimensions can be reliably measured and examined in relation to narcissistic traits and broader personality functioning.

By integrating psychodynamic theory with contemporary psychometric methodology, the present study contributes to an ongoing effort to bridge idiographic theories of personality organization with nomothetic approaches to psychological measurement. Future research incorporating clinical populations, longitudinal designs, cross-cultural samples, and multimethod assessments will be important for determining whether these self-structural dimensions demonstrate clinical relevance, developmental significance, and associations with treatment-related processes.

### 5.5 Exploratory self-structural configurations within the SOSS dimensional space

Beyond examining latent factor structure and dimensional associations, the present study further explored whether individuals could be empirically grouped according to their positions within the SOSS conceptual space. The cluster analysis identified four relatively distinct configurations based on the combined distribution of Self-Cathexis and Self-Space Need. These configurations suggest that individuals may differ not only in the absolute level of specific self-related characteristics but also in how different aspects of self-organization are combined.

The emergence of multiple configurations is consistent with contemporary dimensional perspectives on personality, which emphasize that psychological functioning often reflects patterns of interacting characteristics rather than isolated traits or discrete categories (57, 58). From this perspective, the four-cluster solution provides an exploratory representation of how self-investment and relational-developmental needs may be jointly organized across individuals.

Importantly, these clusters should not be interpreted as categorical personality types or clinical profiles. The moderate silhouette coefficient indicates that the identified groups represent meaningful but not sharply separated configurations. Rather than implying discrete boundaries between forms of self-organization, the clustering results illustrate how continuous variations in Self-Cathexis and Self-Space Need may combine into different patterns within a broader self-structural space.

The concept of self-density provides a theoretical heuristic for interpreting these configurations. Specifically, self-density was proposed as a conceptual descriptor of the relative relationship between psychological investment directed toward the self and the developmental-relational space required for maintaining self-organization. However, this concept should be understood as a theoretical metaphor rather than a directly calculated psychometric parameter in the present study. Future research using longitudinal and clinical samples may further examine whether different self-structural configurations are associated with meaningful differences in personality development, interpersonal functioning, or psychological adjustment.

### 5.6 Implications for psychological assessment and future research

The present findings have several implications for psychological assessment and future research on self-structure and personality functioning. First, the SOSS provides a theoretically informed yet empirically testable framework for assessing self-related processes beyond traditional trait-based approaches. Existing measures of narcissism and personality often focus primarily on observable characteristics or regulatory tendencies, whereas the SOSS was developed to capture broader patterns of self-organization involving self-investment, relational experience, and self-cohesion. In addition to supporting the proposed multidimensional structure, the SOSS demonstrated satisfactory internal consistency in the validation sample, with reliability supported by both Cronbach’s alpha and McDonald’s omega coefficients. Furthermore, measurement invariance analyses indicated that the four-factor structure showed comparable measurement properties across gender groups, providing preliminary support for the applicability of the SOSS across male and female participants. In Study 1, sensitivity analyses using inverse probability weighting further suggested that potential attrition related to retest participation had minimal influence on temporal stability estimates, strengthening confidence in the robustness of the test–retest reliability findings.

Second, the multidimensional structure identified in the present study may provide a more differentiated perspective for examining individual differences in self-related functioning. Rather than interpreting self-organization as a single continuum, the SOSS allows researchers to examine how distinct facets of self-experience may combine in different patterns. This approach may be particularly useful for future studies investigating developmental processes, personality dynamics, and the relationships between self-structure and interpersonal functioning.

Third, the present findings provide a foundation for extending SOSS research to populations beyond the current nonclinical sample. Future studies should examine whether the identified dimensions and configurations show meaningful associations with clinical constructs, including personality pathology, affective dysregulation, and interpersonal difficulties. However, such clinical interpretations require direct validation using clinical samples, diagnostic assessments, and multimethod approaches rather than being inferred from the present findings alone.

Overall, the SOSS should be considered a preliminary research instrument for investigating self-structural organization. Its potential clinical relevance remains an empirical question that requires further examination through longitudinal, cross-cultural, and clinical research designs.

### 5.7 Limitations and future directions

Several limitations should be considered when interpreting the present findings. First, although the current study included large samples across the development and validation phases, participants were recruited from the general population through an online survey platform, and a substantial proportion of participants were young adults and university students. Therefore, the findings primarily reflect patterns of self-structure in a nonclinical population and should not be directly generalized to individuals with personality disorders or clinically significant narcissistic pathology. Future studies should examine the applicability of the SOSS in more diverse populations, including clinical samples and individuals across different developmental stages.

Second, although the present study examined associations between SOSS dimensions and established measures of narcissistic traits, these associations should not be interpreted as evidence that the SOSS assesses narcissistic pathology itself. The observed relationships with NPI and PNI provide preliminary evidence regarding the relevance of self-structural processes to narcissistic characteristics, but further research incorporating clinical interviews, diagnostic assessments, and multimethod approaches is required to determine whether SOSS dimensions are associated with clinically meaningful personality functioning.

Third, although discriminant validity was generally supported, some indicators of convergent and internal consistency were relatively lower for certain facets. In particular, Vulnerable Self showed comparatively lower AVE estimates and reliability coefficients, suggesting that this facet may capture heterogeneous aspects of self-cohesion vulnerability and relational sensitivity. This pattern is consistent with the complex and multidimensional nature of self-structural processes, in which theoretically broad constructs may encompass multiple related psychological experiences. Future research should examine whether additional items or alternative measurement approaches can further improve the internal coherence of specific facets while preserving their theoretical breadth.

Fourth, the present study relied primarily on self-report assessments and cross-sectional data. Although self-report measures are appropriate for assessing subjective self-experiences, they may be influenced by response biases, self-perception limitations, and shared method variance. Moreover, the cross-sectional design prevents conclusions regarding the developmental trajectories or causal relationships among self-structural processes. Longitudinal research will be necessary to investigate how different aspects of self-organization emerge, interact, and change over time.

Fifth, although inverse probability weighting analyses suggested that attrition had minimal effects on the estimated test–retest reliability coefficients, participants who completed the follow-up assessment differed from non-retained participants in demographic characteristics, including age and gender distribution. Therefore, although the temporal stability estimates appeared robust, replication using larger and more representative longitudinal samples is needed to further establish the generalizability of the SOSS over time.

Finally, although the present study provided evidence for the measurement robustness of the SOSS, further validation is needed across cultural contexts and alternative theoretical models. Notably, all participants recruited in the current research were solely from mainland China, resulting in a complete lack of cross-cultural samples to support the generalizability of the scale’s structural patterns. In particular, future studies may examine whether higher-order representations, alternative latent structures, or network-based approaches provide additional insights into the organization of self-related processes. Such investigations will help clarify the extent to which the SOSS captures general principles of self-organization across populations.

## 6 Conclusion

In conclusion, the present research preliminarily developed and conducted a series of psychometric evaluations for the SOSS, an instrument intended to capture interindividual variations in self-structural organization. Across two sequential empirical studies, the obtained results appeared to lend tentative support to a multi-factorial characterization of self-structure, which encompasses four correlated yet partially distinguishable subdomains: Positive Self, Self-Center, Idealized Identification, and Vulnerable Self. Though the SOSS was theoretically derived based on the dichotomy of Self-Cathexis and Self-Space Need, the current validation outcomes imply that these two constructs may operate as overarching interpretive axes, rather than rigidly defined higher-order latent factors with fixed hierarchical relations. Within the nonclinical samples recruited for this work, the SOSS displayed generally acceptable levels of internal consistency, short-term temporal stability, and partial measurement invariance across gender groups; its discriminant validity and theoretically consistent correlations with grandiose and vulnerable narcissism indicators were also observed to reach modest acceptable standards. By attempting to bridge classic psychodynamic accounts of self-organization and modern psychometric methodologies, the SOSS may offer a tentative empirical framework complementary to conventional trait-focused narcissism inventories for exploring diverse self-relevant psychological processes. Nevertheless, the generalizability and clinical utility of this scale remain to be fully established. Follow-up work adopting longitudinal tracking designs, demographically diverse community cohorts, clinical samples, and cross-cultural comparative investigations will be essential to systematically unpack the developmental implications and potential clinical applicability of the proposed self-structural dimensions.

## Data Availability

The original contributions presented in the study are included in the article/**Supplementary Material**. Further inquiries can be directed to the corresponding authors.
