# Supplementary material for: The construction and psychometric evaluation of the self-structure scale: a bidimensional model of self-structure
Source: Front Psychiatry. 2026 Sep 10;17:1942305. doi: 10.3389/fpsyt.2026.1942305 (PMC13601284; doi:10.3389/fpsyt.2026.1942305)
Supplement: Supplementary file 1 [file Table1.docx]

**Supplementary Material**

Supplementary Material for The Construction and Psychometric Evaluation of the Self-Structure Scale (SOSS)

**Reporting conventions**

Descriptive statistics are reported to 2 decimal places, whereas regression coefficients, effect sizes, and test statistics are reported to 3 decimal places unless additional precision is needed for very small estimates. P values smaller than 0.001 are reported as *p < 0.001.*

**Supplementary Table S1. Item Loadings for the Four-Factor EFA Solution of SOSS**

| Item | Factor | | | |
| --- | --- | --- | --- | --- |
|  | Positive Self | Self-Center | Idealized Identification | Vulnerable Self |
| 1 | .598 |  |  |  |
| 2 | .803 |  |  |  |
| 3 | .716 |  |  |  |
| 4 | .851 |  |  |  |
| 7 | .502 |  |  |  |
| 8 | .667 |  |  |  |
| 9 | .420 |  |  |  |
| 11 | .452 |  |  |  |
| 15 | .362 |  |  |  |
| 10 |  | .404 |  |  |
| 12 |  | .620 |  |  |
| 13 |  | .614 |  |  |
| 14 |  | .526 |  |  |
| 16 |  | .534 |  |  |
| 17 |  | .508 |  |  |
| 18 |  | .583 |  |  |
| 20 |  |  | .445 |  |
| 21 |  |  | .644 |  |
| 22 |  |  | .574 |  |
| 23 |  |  | .627 |  |
| 24 |  |  | .650 |  |
| 26 |  |  |  | .415 |
| 27 |  |  |  | .556 |
| 28 |  |  |  | .594 |
| 31 |  |  |  | .419 |
| 32 |  |  |  | .386 |
| 33 |  |  |  | .440 |
| 34 |  |  |  | .380 |

Note. Exploratory factor analysis was conducted using principal axis factoring with oblimin rotation. Only retained items from the final 28-item version are displayed. Loadings < .30 are suppressed for clarity. Item numbers refer to the preliminary 37-item version of the SOSS.

**Supplementary Table S2. Test-Retest Reliability (Two-Month Interval)**

| Scale/Dimension | n | Time 1 Mean (SD) | Time 2 Mean (SD) | ICC(A,1) | 95%Cl | Pearson r |
| --- | --- | --- | --- | --- | --- | --- |
| SOSS total score | 128 | 91.23 (9.97) | 92.16 (10.62) | .870 | [.819, .907] | .874 |
| Self-Cathexis | 128 | 50.36 (7.31) | 51.95 (7.65) | .794 | [.698, .858] | .811 |
| Self-Space Need | 128 | 40.87 (5.85) | 40.21 (5.92) | .799 | [.725, .854] | .803 |
| Positive Self | 128 | 32.34 (5.01) | 33.10 (4.96) | .738 | [.645, .809] | .745 |
| Self-Center | 128 | 18.02 (4.20) | 18.84 (4.77) | .776 | [.687, .841] | .794 |
| Idealized Identification | 128 | 19.73 (2.73) | 19.27 (2.77) | .668 | [.558, .754] | .675 |
| Vulnerable Self | 128 | 21.14 (4.13) | 20.94 (4.24) | .756 | [.671, .821] | .756 |

Note. ICC(A,1) represents a two-way absolute-agreement, single-measure intraclass correlation coefficient. Pearson correlations are reported as supplementary indices of rank-order consistency. CI = confidence interval; SOSS = Scale of Self Structure.

**Supplementary Table S3. Gender-Stratified Test–Retest Reliability and Direct Tests of Gender Differences**

| Scale/Dimension | Male n | Male ICC [95%Cl] | Female n | Female ICC [95%Cl] | ΔICC, F−M [95% CI] | Bootstrap p | BH-adjusted p |
| --- | --- | --- | --- | --- | --- | --- | --- |
| SOSS total score | 28 | .919[.834, .961] | 100 | .843[.775, .892] | -.076[-.178, .050] | 0.188 | 0.473 |
| Self-Cathexis | 28 | .871[.739, .939] | 100 | .750[.629, .832] | -.121[-.288, .106] | 0.203 | 0.473 |
| Self-Space Need | 28 | .861[.725, .933] | 100 | .783[.693, .849] | -.078 [-.196, .115] | 0.364 | 0.637 |
| Positive Self | 28 | .748[.528, .875] | 100 | .735[.627, .815] | -.014[-.194, .263] | 0.978 | 0.978 |
| Self-Center | 28 | .901[.787, .954] | 100 | .711[.594, .798] | -.190[-.328, -.054] | 0.014 | 0.098 |
| Idealized Identification | 28 | .678[.385, .841] | 100 | .668[.544, .764] | -.010[-.197, .240] | 0.968 | 0.978 |
| Vulnerable Self | 28 | .803[.619, .904] | 100 | .745[.643, .821] | -.058[-.209, .185] | 0.6 | 0.84 |

Note. Gender was coded as 1 = male and 2 = female. ΔICC represents the female-minus-male difference. Confidence intervals and p values for ICC differences were estimated using 2,000 stratified bootstrap samples. Benjamini–Hochberg correction was applied across the seven comparisons.

**Supplementary Table S4 Attrition Analysis and Inverse Probability Weighting Sensitivity Analysis for Study 1 Test–Retest Reliability**

**Panel A. Comparison Between Retained and Attrited Participants**

| **Variable** | **Retained (n=128)** | **Attrited (n=1826)** | **p** | **Hedges’ g / Cramer’s V** |
| --- | --- | --- | --- | --- |
| Age | 22.89 (6.84) | 26.77 (8.68) | <.001 | -0.494 |
| SOSS Total | 3.259 (0.356) | 3.210 (0.412) | 0.141 | 0.126 |
| Self-Cathexis | 3.146 (0.458) | 3.202 (0.481) | 0.185 | -0.119 |
| Self-Space Need | 3.408 (0.488) | 3.220 (0.586) | <.001 | 0.347 |
| Positive Self | 3.595 (0.556) | 3.752 (0.603) | 0.003 | -0.27 |
| Self-Center | 2.570 (0.602) | 2.496 (0.696) | 0.184 | 0.114 |
| Idealized Identification | 3.945 (0.546) | 3.790 (0.687) | 0.003 | 0.25 |
| Vulnerable Self | 3.025 (0.593) | 2.813 (0.669) | <.001 | 0.333 |

**Panel B. IPW Sensitivity Analysis of Test–Retest Reliability**

| **Scale** | **Original ICC(A,1)** | **IPW-adjusted ICC(A,1)** |
| --- | --- | --- |
| OSS Total | 0.871 | 0.88 |
| Self-Cathexis | 0.791 | 0.814 |
| Self-Space Need | 0.796 | 0.804 |
| Positive Self | 0.739 | 0.745 |
| Self-Center | 0.771 | 0.813 |
| Idealized Identification | 0.668 | 0.68 |
| Vulnerable Self | 0.753 | 0.751 |

Note. Retained participants were those who completed the two-month follow-up assessment (n = 128), whereas attrited participants were those who did not complete the retest assessment (n = 1,826). Continuous variables were compared using independent-samples t tests, with Hedges’ g reported as the effect size. Gender distribution was compared using chi-square tests, with Cramer’s V reported as the effect size. Attrition analyses examined whether demographic characteristics and baseline SOSS scores differed between retained and attrited participants. Inverse probability weighting (IPW) sensitivity analyses were conducted based on predicted probabilities of retest participation estimated from logistic regression models including age, gender, Positive Self, Self-Center, Idealized Identification, and Vulnerable Self. IPW-adjusted ICC(A,1) estimates were compared with the original ICC(A,1) estimates to evaluate the robustness of test–retest reliability findings. ICC(A,1) = two-way absolute-agreement, single-measure intraclass correlation coefficient; IPW = inverse probability weighting.

**Supplementary Table S5. Sensitivity analysis of competing measurement models treating SOSS items as continuous indicators**

| Model | χ²(scaled) | df | CFI | TLI | RMSEA (90% CI) | SRMR |
| --- | --- | --- | --- | --- | --- | --- |
| 2-factor CFA | 10423.476 | 349 | 0.629 | 0.598 | .087 [.086, .089] | 0.111 |
| 4-factor CFA | 5432.64 | 344 | 0.813 | 0.794 | .062 [.061, .064] | 0.068 |
| Higher-order Model A | Not estimated | - | - | - | - | - |
| Higher-order Model B | 5979.701 | 346 | 0.793 | 0.773 | .065 [.064, .067] | 0.084 |
| Higher-order Model C | 5528.996 | 344 | 0.809 | 0.79 | .063 [.061, .064] | 0.071 |
| 4-factor ESEM | 2522.857 | 272 | 0.917 | 0.885 | .047 [.045, .048] | 0.029 |

Note. Sensitivity analyses were conducted by treating the five-point Likert-scale SOSS items as continuous indicators and estimating models using maximum likelihood (ML) estimation. CFI = comparative fit index; TLI = Tucker–Lewis index; RMSEA = root mean square error of approximation; CI = confidence interval; SRMR = standardized root mean square residual. Higher-order Model A did not converge and therefore did not yield interpretable fit indices. The pattern of model comparison was comparable to the primary ordinal-indicator analysis, with the four-factor ESEM model demonstrating the best relative fit among the tested models.

**Supplementary Table S6 Measurement invariance analyses of the SOSS across gender groups**

**Panel A. Four-factor ESEM model (primary)**

| **Model** | **χ²** | **df** | **CFI** | **TLI** | **RMSEA (90% CI)** | **SRMR** | **ΔCFI** | **ΔRMSEA** | **ΔSRMR** |
| --- | --- | --- | --- | --- | --- | --- | --- | --- | --- |
| Configural invariance | 3809.901 | 552 | 0.887 | 0.845 | .063 [.061, .064] | 0.035 | — | — | — |
| Metric invariance | 3008.564 | 700 | 0.955 | 0.951 | .042 [.040, .043] | 0.038 | 0.068 | −.021 | .003 |
| Scalar invariance | 2878.842 | 728 | 0.958 | 0.956 | .039 [.038, .041] | 0.038 | 0.003 | −.002 | .000 |

Note. Measurement invariance analyses were conducted using the four-factor exploratory structural equation modeling (ESEM) solution of the Scale of Self Structure (SOSS) with WLSMV estimation. Configural invariance tested whether the same factor structure was supported across male and female groups. Metric invariance constrained factor loadings to equality across groups, whereas scalar invariance additionally constrained item thresholds to equality. Changes in robust fit indices (ΔCFI, ΔRMSEA, and ΔSRMR) were used to evaluate invariance. Following commonly used practical criteria (ΔCFI ≤ .010, ΔRMSEA ≤ .015, and ΔSRMR ≤ .030 for metric invariance; ΔCFI ≤ .010, ΔRMSEA ≤ .015, and ΔSRMR ≤ .010 for scalar invariance), both metric and scalar invariance were supported.

**Panel B. Four-factor CFA model (supplementary)**

| **Model** | **χ²** | **df** | **CFI** | **TLI** | **RMSEA (90% CI)** | **SRMR** | **ΔCFI** | **ΔRMSEA** | **ΔSRMR** |
| --- | --- | --- | --- | --- | --- | --- | --- | --- | --- |
| Configural invariance | 3809.901 | 552 | 0.887 | 0.845 | .063 [.061, .064] | 0.035 | — | — | — |
| Metric invariance | 3008.564 | 700 | 0.955 | 0.951 | .042 [.040, .043] | 0.038 | 0.068 | −.021 | .003 |
| Scalar invariance | 2878.842 | 728 | 0.958 | 0.956 | .039 [.038, .041] | 0.038 | 0.003 | −.002 | .000 |

Note. Although metric and scalar invariance criteria were statistically satisfied for the four-factor CFA model, the absolute fit of the CFA solution was relatively weaker than the ESEM solution. Therefore, CFA invariance results were interpreted as supplementary evidence, whereas the ESEM invariance results were considered more consistent with the retained measurement model.

**Supplementary Table S7. Internal Consistency Reliability of the SOSS in Study 2**

| Scale | Number of Items | Cronbach’s α | McDonald’s ω | 95% CI for ω |
| --- | --- | --- | --- | --- |
| SOSS Total | 28 | 0.795 | 0.859 | [.849, .868] |
| Self-Cathexis | 14 | 0.811 | 0.846 | [.845, .864] |
| Self-Space Need | 14 | 0.744 | 0.727 | [.723, .779] |
| Positive Self |  | 0.841 | 0.821 | [.809, .842] |
| Self-Center |  | 0.726 | 0.651 | [.678, .727] |
| Idealized Identification |  | 0.698 | 0.631 | [.637, .687] |
| Vulnerable Self |  | 0.657 | 0.592 | [.560, .658] |

Note. Cronbach’s α and McDonald’s ω coefficients were calculated to evaluate internal consistency reliability of the Scale of Self Structure (SOSS). McDonald’s ω coefficients and their 95% confidence intervals were estimated based on the retained four-factor measurement structure using bootstrap procedures. Higher values indicate greater internal consistency reliability.

**Supplementary Table S8. Inter-factor correlations among the four-factor SOSS structure**

|  | 1 | 2 | 3 | 4 |
| --- | --- | --- | --- | --- |
| 1. Positive Self | — |  |  |  |
| 2. Self-Center | .267*** | — |  |  |
| 3. Idealized Identification | .059** | .323*** | — |  |
| 4. Vulnerable Self | -.168*** | .320*** | .397*** | — |

Note. **p<.05, **p<.01, ***p<.001.* Pearson correlations were calculated using the combined sample from Samples 3 and 4 (N = 3,805). Positive Self, Self-Center, Idealized Identification, and Vulnerable Self represent the four first-order factors of the SOSS.

**Supplementary Table S9. Average variance extracted and square roots of average variance extracted for the four-factor SOSS structure**

|  | Positive Self | Self-Center | Idealized Identification | Vulnerable Self |
| --- | --- | --- | --- | --- |
| AVE | .460 | .324 | .407 | .260 |
| Square root of AVE | .678 | .569 | .638 | .510 |

Note. AVE = average variance extracted; SOSS = Scale of Self Structure. AVE values were calculated from standardized confirmatory factor analysis loadings. Average variance extracted and square roots of average variance extracted for the four-factor SOSS structure

**Supplementary Table S10. Analysis of Variance (ANOVA) for Differences Across the Four-Cluster Solution in the SOSS Dimensional Space**

| Dimension  (z score) | Mean Square | *df* | Error Mean Square | *df (Error)* | F | p |
| --- | --- | --- | --- | --- | --- | --- |
| Self-Cathexis | 2237 | 3 | 1567 | 3801 | 1808 | <.001 |
| Self-space need | 2448 | 3 | 1356 | 3801 | 2289 | <.001 |

Note. SOSS = Scale of Self Structure. ANOVAs were conducted to examine whether the four clusters differed significantly on the two theoretically derived composite dimensions, Self-Cathexis and Self-Space Need. Scores were standardized before clustering analyses. df = degrees of freedom.

**Supplementary Figure S1. Standardized factor loadings and cross-loadings from the four-factor target-rotated ESEM solution of the SOSS**

**Supplementary Figure S2. Split-half scatterplot of the four-cluster solution (Random 50% Subsample)**

**Supplementary Material A. Final English Version of the Scale of Self Structure (SOSS)**

Instructions:

Below are statements about self-perception. There are no right or wrong answers. Please select the option that best reflects your true experience:

1 = Completely Disagree

2 = Mostly Disagree

3 = Uncertain

4 = Mostly Agree

5 = Completely Agree

1. Overall, I hold myself in high regard.
2. I never belittle myself under any circumstances.
3. My self-perception remains stable regardless of external changes.
4. I consistently affirm my self-worth: "I am exceptional."
5. Intrinsic satisfaction suffices when I engage in extensive work.
6. I maintain clear, unwavering life goals and plans.
7. Most acquaintances perceive me as having distinct personality traits.
8. Others describe me as stubborn or inflexible.
9. My self-view has remained consistent over years, regardless of circumstances.
10. Some characterize me as self-centered, though I rarely take offense.
11. I believe I deserve special privileges in many situations.
12. Failures reflect situational misfortune rather than personal inadequacy.
13. I trust my capabilities will eventually be recognized ("Gold shines in time").
14. In domains where I excel, I doubt others’ superiority.
15. I expect close associates to fully commit to my endeavors.
16. I frequently delegate personal arrangements to those around me.
17. Validation from significant others is emotionally crucial to me.
18. Acceptance by authority figures feels fortuitous.
19. Public attention enhances my self-perception.
20. I desire recognition of my achievements from valued individuals.
21. Collaborating with competent peers bolsters my confidence in challenging tasks.
22. Feeling undervalued provokes irritability or anger.
23. Solitude evokes an inexplicable sense of emptiness.
24. Comparing myself to superior others induces helplessness.
25. I actively seek external evaluations of myself.
26. I strive to preserve harmonious relationships ("Harmony first").
27. I conceal past embarrassments from others.
28. I prefer supportive roles over leadership positions in collaborative work.

**Scoring Protocol:**

Dimensions/Factors: Higher scores indicate stronger endorsement.

Self-Cathexis Dimension:

Positive Self: Items 1, 2, 3, 4, 5, 6, 7, 9, 13

Self-Center: Items 8, 10, 11, 12, 14, 15, 16

self-space need Dimension:

Idealized Identification: Items 17, 18, 19, 20, 21

Vulnerable Self: Items 22, 23, 24, 25, 26, 27, 28

Dimension Composition:

Self-Cathexis: Items 1–16

self-space need: Items 17–28
